# RNA-sequencing reveals early, dynamic transcriptome changes in the corollas of pollinated petunias

**DOI:** 10.1186/s12870-014-0307-2

**Published:** 2014-11-18

**Authors:** Shaun R Broderick, Saranga Wijeratne, Asela J Wijeratn, Laura J Chapin, Tea Meulia, Michelle L Jones

**Affiliations:** Department of Horticulture and Crop Science, The Ohio Agricultural Research and Development Center, The Ohio State University, 1680 Madison Ave, Wooster, OH 44691 USA; Molecular and Cellular Imaging Center, The Ohio Agricultural Research and Development Center, The Ohio State University, 1680 Madison Ave, Wooster, OH 44691 USA

**Keywords:** RNA-seq, WGCNA, *de novo* assembly, String, KEGG, Trinity, Autophagy, Calcium signaling, Ethylene, Petal senescence

## Abstract

**Background:**

Pollination reduces flower longevity in many angiosperms by accelerating corolla senescence. This response requires hormone signaling between the floral organs and results in the degradation of macromolecules and organelles within the petals to allow for nutrient remobilization to developing seeds. To investigate early pollination-induced changes in petal gene expression, we utilized high-throughput sequencing to identify transcripts that were differentially expressed between corollas of pollinated *Petunia × hybrida* flowers and their unpollinated controls at 12, 18, and 24 hours after opening.

**Results:**

In total, close to 0.5 billion Illumina 101 bp reads were generated, *de novo* assembled, and annotated, resulting in an EST library of approximately 33 K genes. Over 4,700 unique, differentially expressed genes were identified using comparisons between the pollinated and unpollinated libraries followed by pairwise comparisons of pollinated libraries to unpollinated libraries from the same time point (i.e. 12-P/U, 18-P/U, and 24-P/U) in the Bioconductor R package DESeq2. Over 500 gene ontology terms were enriched. The response to auxin stimulus and response to 1-aminocyclopropane-1-carboxylic acid terms were enriched by 12 hours after pollination (hap). Using weighted gene correlation network analysis (WGCNA), three pollination-specific modules were identified. Module I had increased expression across pollinated corollas at 12, 18, and 24 h, and modules II and III had a peak of expression in pollinated corollas at 18 h. A total of 15 enriched KEGG pathways were identified. Many of the genes from these pathways were involved in metabolic processes or signaling. More than 300 differentially expressed transcription factors were identified.

**Conclusions:**

Gene expression changes in corollas were detected within 12 hap, well before fertilization and corolla wilting or ethylene evolution. Significant changes in gene expression occurred at 18 hap, including the up-regulation of autophagy and down-regulation of ribosomal genes and genes involved in carbon fixation. This transcriptomic database will greatly expand the genetic resources available in petunia. Additionally, it will guide future research aimed at identifying the best targets for increasing flower longevity by delaying corolla senescence.

**Electronic supplementary material:**

The online version of this article (doi:10.1186/s12870-014-0307-2) contains supplementary material, which is available to authorized users.

## Background

The longevity of individual flowers is genetically programmed to allow for efficient reproduction while limiting energy costs associated with maintaining the petals [1,2]. In many angiosperms, pollination reduces flower longevity and initiates global gene expression changes that lead to flower senescence [3,4]. Pollination-induced senescence of the corolla allows for nutrients to be recycled from the petals to the developing ovary [2,5]. In petunias, ethylene biosynthesis is induced by pollination, and the application of exogenous ethylene accelerates senescence [6]. Ethylene in wild type petunias can be measured from pollinated styles within an hour after pollination. This initial ethylene production is not sufficient to induce corolla senescence, but is followed by ethylene biosynthesis in the corolla, which then induces petal wilting [4,7,8]. In an effort to extend flower longevity, transgenic approaches have been utilized to alter ethylene perception in petunia. These experiments have created ethylene insensitive petunia flowers that last approximately twice as long as wild type flowers and do not undergo accelerated senescence after pollination [4,6,9,10].

Pollen is thought to contain a signaling factor(s) that triggers petal senescence in ethylene-sensitive species [11]. Relatively large amounts of 1-aminocyclopropane-1-carboxylic acid (ACC) and auxin are found in petunia pollen, but experimental evidence has shown that only excessive amounts of these substances are able to increase ethylene production and accelerate flower senescence [11,12]. Other factors such as short-chain fatty acids and changes in electrical potential may play a larger role in pollination-induced petal senescence, either by acting as a signaling factor or by increasing ethylene sensitivity [11,13]. While pollination induces ethylene production and leads to senescence in ethylene-sensitive flowers, it remains unclear how pollination is linked to ethylene biosynthesis. Rather than blocking downstream ethylene-induced responses to delay flower senescence, inhibiting pollination signals that lead to ethylene biosynthesis may provide an alternative means of extending flower longevity.

Transcriptomic approaches, including microarrays and RNA-sequencing (RNA-seq), have been used to profile gene expression changes during flower petal development and senescence in multiple species [14–22]. A large percentage of the genes that are up-regulated during senescence encode enzymes involved in degradation and transport. The systematic degradation of proteins, nucleic acids, lipids, and cell wall components allows for the remobilization of sugars and other nutrients before the death of the petal cells [23]. A suppressive subtractive hybridization experiment in *Alstroemeria* flowers showed that genes involved in cell wall synthesis, protein synthesis, metabolism, and signaling were most abundant in the petals of younger flowers, while those involved in macromolecule breakdown were highest at the later stages [20]. Pollination-induced senescence involves similar processes and can reduce flower longevity of *Ophrys* (orchid) to five or six days. In orchid labella, genes involved in macromolecular breakdown, stress and defense, and nutrient remobilization are differentially expressed after pollination. Floral scent and pigment genes are down-regulated by two days after pollination [19].

While microarrays have been utilized to study gene expression changes in petunia [17,18], to our knowledge, genome-wide expression profiling using RNA-sequencing (RNA-seq) has not been performed in petunia flowers. Microarrays are able to measure gene expression changes, but are limited by the availability of Expressed Sequence Tags (ESTs). Additionally, highly expressed genes can saturate the microarrays and reduce the accuracy of gene expression data, especially for lower expressed genes. RNA-seq experiments can provide a global overview of gene expression during corolla senescence without any *a priori* genetic data. The recent reductions in sequencing costs have made this technology more readily accessible to researchers. RNA-seq is particularly useful for identifying genes and their isoforms, and it can measure gene expression levels that have more than an 8,000-fold difference [24,25].

This experiment was designed to profile early gene expression changes in petunia corollas following pollination, with the goal of identifying the signaling pathways that are involved in initiating corolla senescence. Another objective was to generate an assembled and annotated RNA-seq transcriptome for petunia corollas. Data from this experiment will provide a valuable addition to the molecular resources available for petunia. This research will guide the future selection of promising candidate genes for extending flower longevity by delaying corolla senescence.

## Results and discussion

### Pollen tube growth and ethylene biosynthesis of post-anthesis petunia flowers

Pollination accelerates the senescence of petunia flowers. Inducing flower senescence by pollination synchronizes the senescence program and allows for the collection of corollas that are at a very similar stage of senescence [26]. A characterization of pollen tube growth, ethylene production, and visual senescence symptoms in *Petunia × hybrida* ‘Mitchell Diploid’ was conducted to identify the best time points for RNA-seq library construction. The goal was to identify genes and pathways involved in early senescence signaling within the corolla, so time points before fertilization, climacteric ethylene production from the petals, and visual corolla wilting were desired.

**Figure 1:**
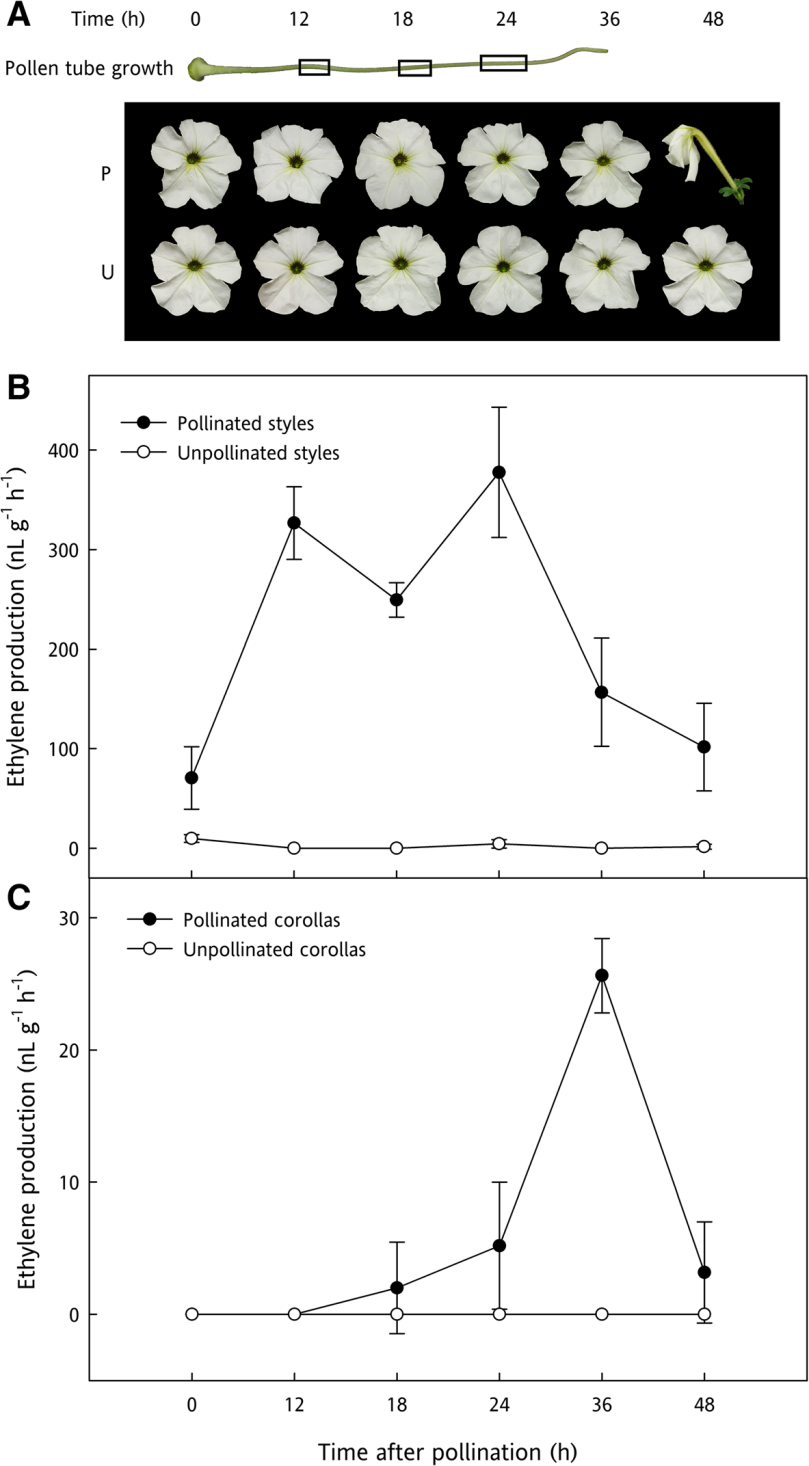
**Characterization of pollinated ‘Mitchell Diploid’ petunia flowers. (A)** Pollen tube growth and petal wilting after pollination. Black rectangles overlaid on the style mark the pollen tube growth at 12, 18, and 24 hap (*n* = 4). Rectangle width is proportional to ± SD. **(B)** Ethylene production of unpollinated and pollinated styles. **(C)** Ethylene production of corollas from pollinated and unpollinated flowers (*n = *6). Mean ethylene levels were used to create the line graphs, and the error bars represent ± SD. Vertical alignment of the time points are consistent for the entire figure.

Pollinated corollas were slightly less turgid (i.e. soft to the touch) at 36 hap and were visibly wilted by 48 hap. Corollas of pollinated flowers from 0 – 24 hours after pollination (hap) were morphologically indistinguishable from each other and from unpollinated flowers of the same age. Previous studies have shown that unpollinated flowers are not wilted until around 192 h [27]. Pollen tube growth was measured at various times after pollination. Pollen tubes maintained a relatively steady, linear growth rate and reached the end of the style after 24 hap, but before 36 hap (Figure 1A). Ethylene biosynthesis from styles and corollas was measured separately at specific times after pollination. In the initial measurements, ethylene production could be detected from pollinated styles, and ethylene peaked at 12 and 24 hap, with a slight decline at 18 hap. Ethylene production sharply declined at 36 and 48 hap (Figure 1B). In pollinated corollas, ethylene was first detectable at 18 hap, though at very low levels (2.3 nl g^−1^ h^−1^). Ethylene production peaked at 36 hap, followed by a sharp decline at 48 hap (Figure 1C). Previous studies have demonstrated that ethylene, *ACC synthase,* and ACC increase within the first hap, predominantly in the stigma [8,28]; however, this initial ethylene production (within the first seven hours) is not sufficient to induce petal wilting. Pollination, therefore, requires additional factors to induce ethylene production in the corollas that leads to petal senescence [8].

### Petunia corolla EST library construction and evaluation

**Figure 2:**
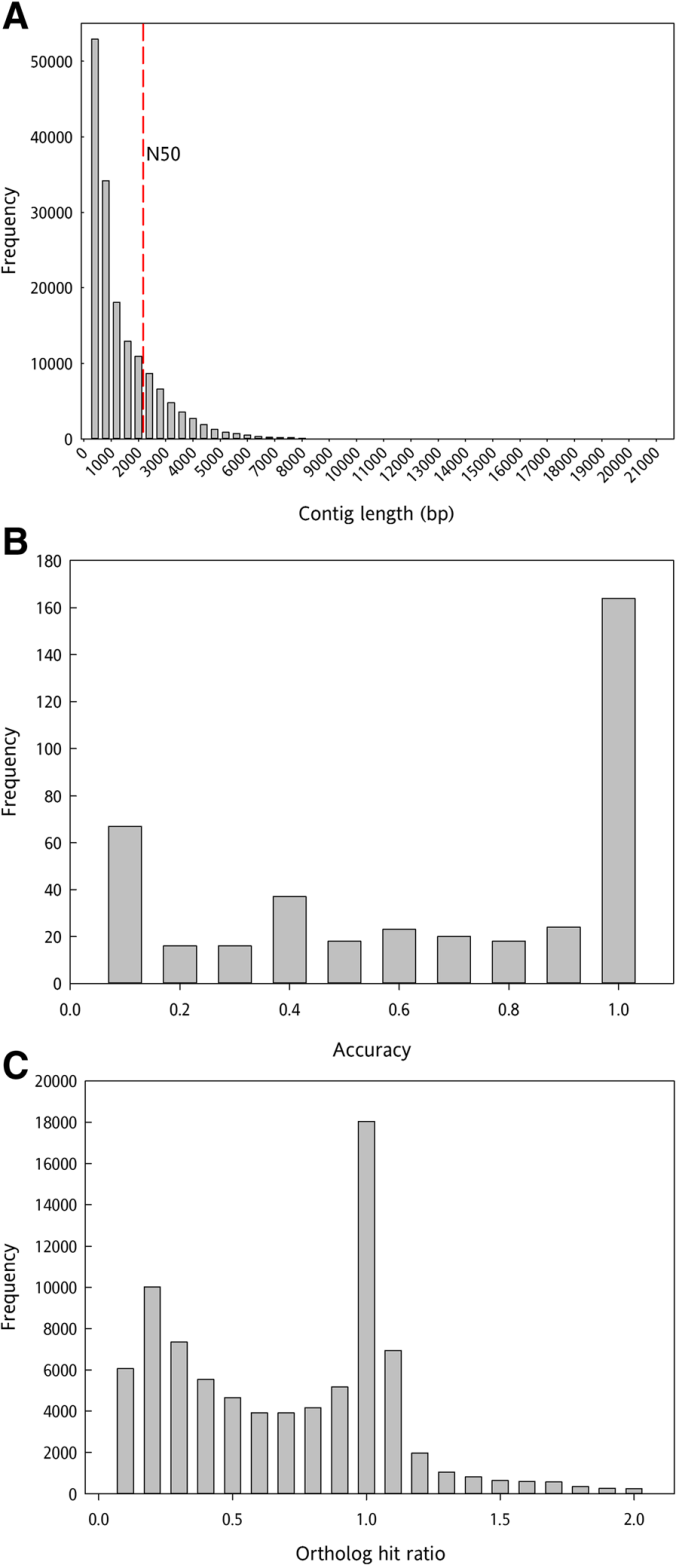
**Transcriptome assembly length and quality. (A)** Contig length distribution. The N50 of 2,181 bp is designated with a dotted vertical line. **(B)** Sequence similarity distribution of the assembled contigs to 404 full-length *Petunia × hybrida* coding sequences from GenBank. **(C)** Ortholog hit ratio distribution between the assembled contigs and the tomato ITAG2.3 protein database.

Strand-specific RNA-sequencing libraries were constructed from corolla mRNA of unpollinated and pollinated flowers at 12, 18, and 24 hours after flower opening. Using the Illumina HiSeq platform, we generated a total of 488,762,314 paired-end reads that were 101 bp in length from 18 libraries. Reads per library ranged from 11,502,467 to 47,030,266, with a mean of 27,153,462 (Table 1). After preprocessing and quality trimming, the remaining 471,116,383 paired-end reads were used for *de novo* transcriptome assembly. We chose Trinity for *de novo* assembly because it has been shown to be more accurate than other programs, including Trans-ABySS and SOAPdenovo-trans [29,30]. A total of 161,974 contigs were generated using Trinity [31], and they had an N50 of 2,181 bp (Figure 2A).

**Table 1:** **Illumina HiSeq read processing and mapping results from RNA-seq petunia corolla libraries**

	**Treatment**	**Hap**	**Biological replicate**	**Sequencing lane**	**Index**	**Total reads**	**Processed reads**	**Unique counts**
1	Pollinated	12	1	6	ATCACG	30832201	30141993	33039451
2	Pollinated	18	1	6	TTAGGC	25490151	25372935	29427524
3	Pollinated	24	1	6	ACAGTG	25117567	24877954	29517441
4	Unpollinated	12	1	6	CGATGT	30268558	28834631	30281445
5	Unpollinated	18	1	6	TGACCA	24763272	24641813	29090675
6	Unpollinated	24	1	6	GCCAAT	24371192	23570895	27670018
7	Pollinated	12	2	7	ATCACG	28373851	26976578	27909905
8	Pollinated	18	2	7	TTAGGC	11502467	11464155	13421728
9	Pollinated	24	2	7	ACAGTG	27673714	27592458	31866197
10	Unpollinated	12	2	7	CGATGT	29698309	28407270	29408647
11	Unpollinated	18	2	7	TGACCA	23585772	23489502	27520959
12	Unpollinated	24	2	7	GCCAAT	47030266	41469932	48764300
13	Pollinated	12	3	8	ATCACG	27800105	25311931	26148002
14	Pollinated	18	3	8	TTAGGC	24646831	24567933	28698650
15	Pollinated	24	3	8	ACAGTG	24659230	23812228	27191584
16	Unpollinated	12	3	8	CGATGT	23101938	21107740	21750346
17	Unpollinated	18	3	8	TGACCA	22719350	22563678	26873758
18	Unpollinated	24	3	8	GCCAAT	37127540	36912757	43177652
	All libraries					488762314	471116383

To evaluate the accuracy of the assembly, the contigs were compared to 404 complete *Petunia × hybrida* coding sequences (CDS) available in GenBank (www.ncbi.nlm.nih.gov). From the GenBank-obtained sequences, 164 (41%) were 90-100% identical to the *de novo* assembled contigs (Figure 2B). The ortholog-hit ratio (OHR) [32] was calculated using the *Solanum lycopersicum* ITAG2.3 protein database, and 44% of the contigs had an OHR between 0.8 and 1.2 (Figure 2C). Together, these comparisons indicate that the *de novo* assembly was robust and accurate.

To generate an EST library, the 162 K contigs were screened for ORFs using TransDecoder, and 37,939 contigs contained putative ORFs larger than 100 amino acids. Additionally, we added 619 contigs that had an OHR greater than 0.8 and did not share the same component identification number that was assigned by Trinity. This was done to prevent removal of contigs that had a putative *S. lycopersicum* ortholog. Finally, contigs of high similarity to each other (threshold of 90%) were removed using CD-HIT-EST. This threshold was selected to increase the number of uniquely mapped reads during expression analysis, and resulted in an expressed sequenced tagged (EST) library of 33,292. A total of 26,006 genes met specific annotation thresholds and were successfully annotated using Blast2GO. Our data represents the first RNA-seq generated transcriptome from petunia corollas.

### Differential gene expression identifies many pollination-associated gene changes

**Figure 3:**
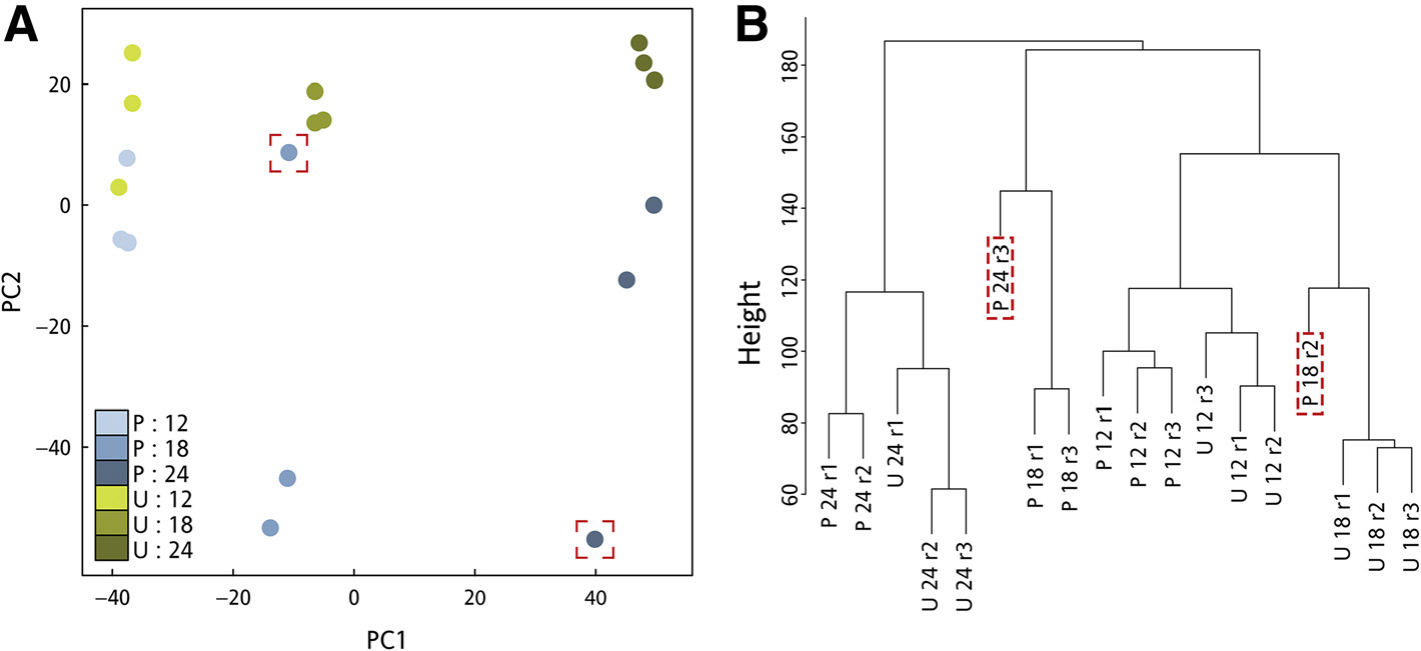
**Tests for outlying RNA-seq libraries. (A)** Principle component analysis plot of the RNA-seq libraries. Green circles correspond to unpollinated libraries and blue circles correspond to pollinated libraries. **(B)** Average linkage hierarchical cluster tree of the 18 RNA-seq libraries. Red, dashed boxes are placed around outlying libraries.

Expression data was generated by aligning the preprocessed, quality-trimmed reads to the EST library. Approximately 84% of the reads from all libraries mapped to the EST library. We used the principle component analysis (PCA) function within the R package DESeq2 [33] and the average linkage cluster tree analysis within the weighted gene network correlation analysis (WGCNA) R package [34,35] to screen for outlying libraries (Figure 3). PCA revealed that the libraries were segregated horizontally (PC1) based on the time of sample collection. Vertical segregation (PC2) occurred between pollinated and unpollinated samples at 18 and 24 hap. The linkage cluster tree revealed that libraries P18 r2 and P24 r3 did not group with their corresponding biological replicates. The correlation between the biological replicates of the libraries was calculated and visualized using scatterplots. All biological replicates had a strong correlation (*R*^*2*^ value above 0.9) except for libraries P18 r2 and P24 r3 (Additional file 1). Based on these results, outlying libraries P18 r2 and P24 r3 were removed from further analysis. Library P18 r2 had 11.5 M reads, which is 58% lower than the average library reads. Reduced sequencing depths in RNA-seq experiments result in less reliable gene expression data, especially for low-expressed genes [25]. The other outlying library (P24 r3) had good sequencing coverage, but did not group with the other pollinated 24 hour replicates. This may have resulted from differences in pollen load, pollen viability, or stigma damage during emasculation [36,37].

**Figure 4:**
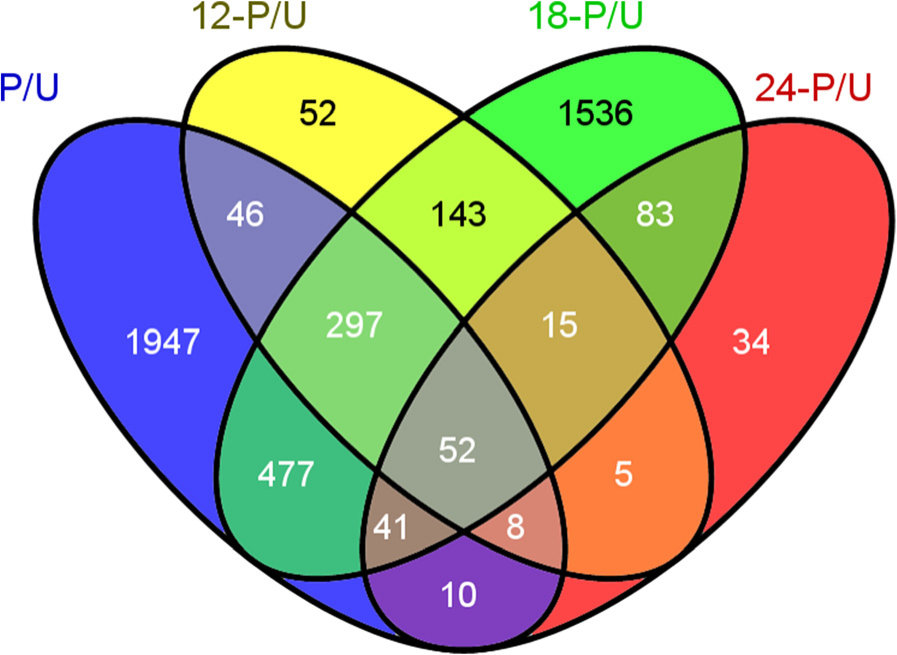
**VENN diagram of differentially expressed genes.** A VENN diagram displaying the overlapping differentially expressed genes identified from pairwise comparisons of pollinated and unpollinated libraries at all time points (P/U) and of pollinated and unpollinated libraries at 12, 18, and 24 h (12-P/U, 18-P/U and 24-P/U, respectively).

DESeq2 was used to identify significant pollination-associated gene changes in petunia corollas. Using normalized count data, 2,878 significant (FDR <0.05) differentially expressed genes were identified after comparing the pollinated and unpollinated treatments (P/U). Additionally, pairwise comparisons between libraries of the same time points were made (e.g. 12 hour pollinated versus 12 hour unpollinated; 12-P/U). The 12-P/U list contained 618 differentially expressed genes, 18-P/U had 2,644, and 24-P/U had 248 (Additional file 2). A total of 4,746 non-redundant (i.e. genes that were differentially expressed in more than one pairwise comparison were only counted once), pollination-associated genes were identified from these pairwise comparisons (Figure 4). These data showed that thousands of gene changes occurred well before the corollas displayed any visual symptoms of senescence (i.e. wilting) and before the pollen tubes have reached the base of the style (Figure 1A).

The total number of gene changes demonstrates the complex, dynamic, and orchestrated processes of initiating petal senescence in petunia. These findings are in line with other flower development studies. For example, RNA-seq data from developing *Chimonanthus praecox* (wintersweet) flowers had 2,706 differentially expressed genes between bud and open flowers and 1,466 between open and senescent flowers [14]. More than 5,400 differentially expressed genes were identified in *Rosa chinensis* between open and senesced flowers [38]. A microarray experiment in orchid (*Ophrys fusca*) compared pollinated and unpollinated labella and found that 8.2% of the printed cDNA clones were differentially expressed within 48 hours after pollination. These gene changes occurred long before visual cues of senescence were visualized at 5 to 6 days after pollination [19]. Together these data demonstrate the highly dynamic nature of transcriptomic data in senescing flowers. Similarly, transcriptomic studies in leaves have identified thousands of genes that show either increased or decreased expression during leaf senescence [39,40].

### Weighted gene correlation network analysis identified three pollination-specific modules

**Figure 5:**
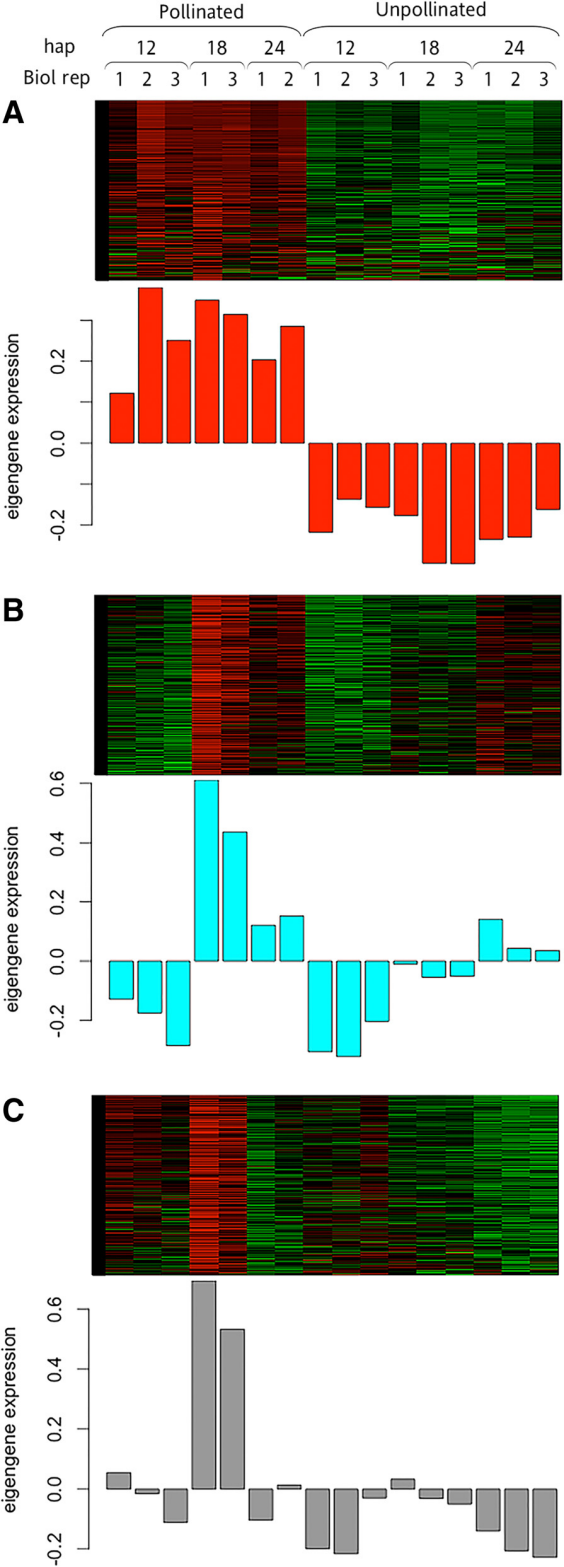
**Heatmaps and eigengene expression patterns for pollination-specific WGCNA modules.** Heatmap and eigengene expression profile across each library for **(A)** Module I (red) **(B)** Module II (cyan) and **(C)** Module III (grey60). Treatment, collection times (hap), and biological replicate numbers (Biol rep) are indicated above each column in the heatmap and eigengenes profiles. Columns are vertically aligned for all three modules.

**Figure 6:**
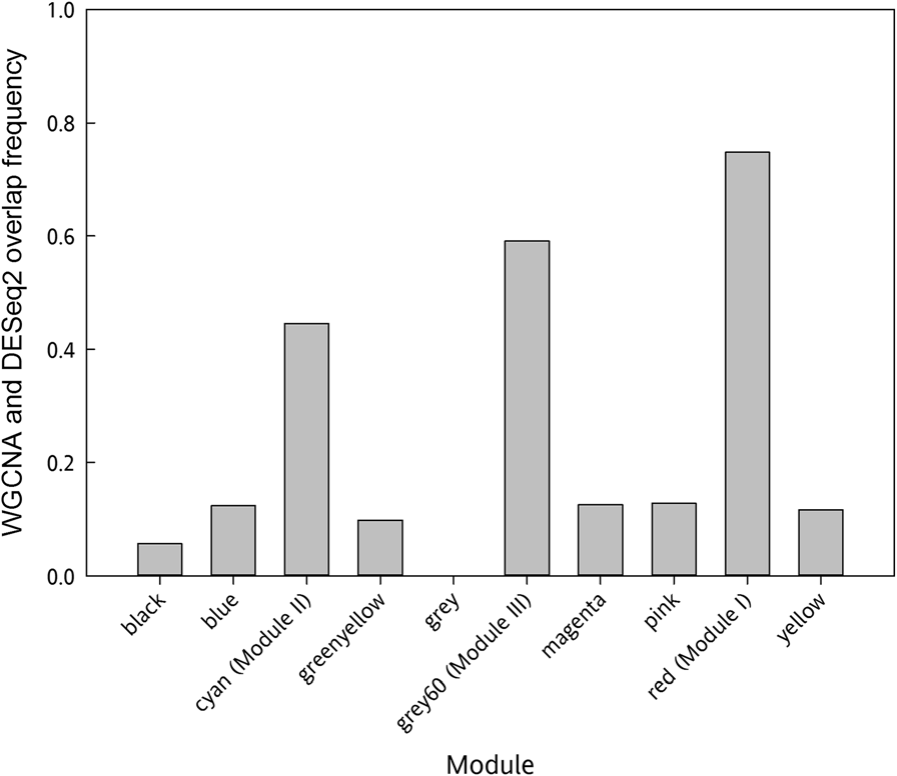
**Frequency of overlapping contigs between DESeq2 and WGCNA.** The bars represent the ten WGCNA modules and correspond to the frequency of overlapping contigs to the sequences that were obtained from the DESeq2 analysis. Cyan (Module II), grey60 (Module III) and red (Module I) have pollination-specific expression patterns.

The differential gene expression analyses identified significant changes in thousands of genes after pollination. We hypothesized that many pollination-associated genes may be acting together in a network to regulate senescence in the corolla. Genes that form protein complexes often share similar expression patterns [41]. To test this hypothesis, WGCNA was used to identify gene clusters (modules) that have highly correlative expression patterns. With a stringency threshold of 0.75, a total of 10 modules were identified from petunia corollas using WGCNA (Additional file 3). Three of these modules had expression patterns that were associated with pollination (i.e. changes in expression profiles appeared in only one treatment for at least one time point), and these included red (Module I), cyan (Module II), and grey60 (Module III). Heatmaps of the modules were generated to visualize the gene expression patterns over time (Figure 5). Module I had increased gene expression across all times points (12, 18 and 24 h) in corollas of pollinated flowers. This module had 1,303 genes, 75% of which also belong to the DESeq2 P/U differentially expressed gene list (Figure 6). Module II consisted of 780 contigs and was the smallest. This module’s expression (i.e. eigengene) was similar at 12 and 24 h, but expression was up-regulated at 18 h in pollinated corollas. It had 348 genes (45%) in common with the 18-P/U differentially expressed genes. The largest of the modules was Module III, containing 1,359 genes. It was similar to Module II (Figure 5B, C) in expression patterns and contained 803 (59%) genes in common with the 18-P/U differentially expressed genes (Figure 6).

The WGCNA and DESeq2 analyses both identified two main expression patterns (i.e. genes that were differentially expressed in pollinated corollas and genes that were differentially expressed at 18 hap) when comparing corollas from pollinated and unpollinated flowers at the same developmental age. Pollination induced changes in gene expression that occurred prior to fertilization and ethylene biosynthesis in the corollas. After pollination, it took more than 24 h for pollen tubes to reach the bottom of the style (Figure 1A). Therefore, a signal(s) must precede fertilization to elicit the expression changes in the corolla that lead to accelerated petal senescence. Pollination signaling may involve ACC, auxin, ethylene, short-chain fatty acids, or electrical pulses [13,36,42]. Although ethylene production did not peak until 36 hap in corollas, the styles produced ethylene within the first hour after pollination and continued for 48 h. Inhibiting ethylene production or perception in the style with aminoethyoxyvinylglycine (AVG) or diazocyclopentadiene (DACP), respectively, prevents pollination-induced corolla senescence [8,43]. These results suggest that ethylene signaling within the gynoecium is required for the corollas to respond to pollination. However, the ethylene from pollinated styles that are immediately severed from the flower, but left in the corolla, is not sufficient to accelerate senescence [11], suggesting that additional factors must be transmitted to the corolla to induce senescence. Wounding also results in elevated ethylene production from petunia stigmas, and at 17 hours after the stigma wounding, petal wilting can no longer be delayed by removing the damaged stigmas [44]. This suggests that the necessary signals for stigma-induced, flower senescence are in place within the first 17 hours after stigma wounding. Short-chain fatty acids that are produced in the gynoecium and transported to the corolla within 12 h of pollination have been shown to increase ethylene sensitivity in corollas, and this may be a component of the pollination signaling [11,13].

### Validation of RNA-seq data by quantitative PCR

**Figure 7:**
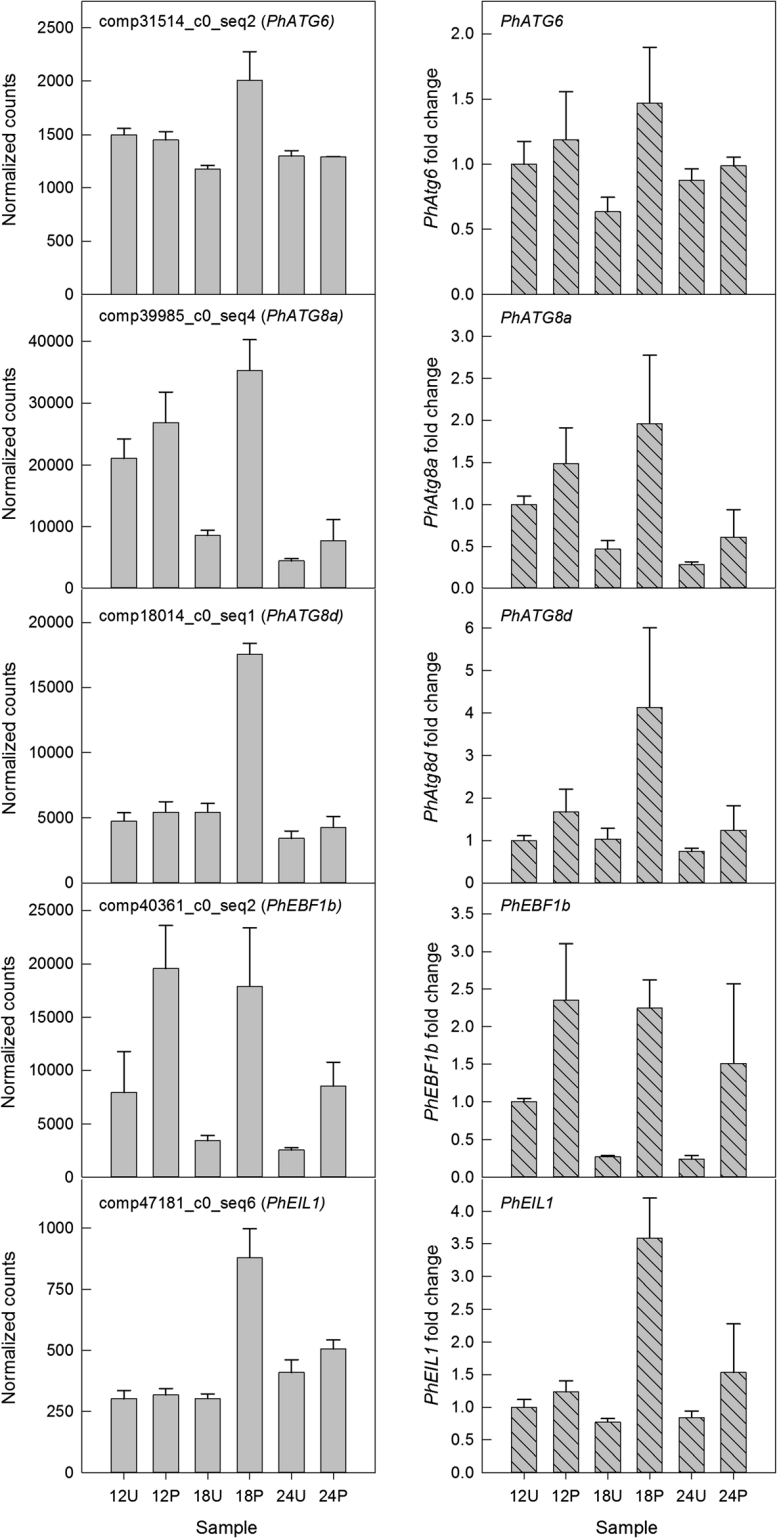
**Quantitative PCR validation of RNA-seq data.** Five genes were selected for qPCR analysis to confirm expression patterns. The left column of graphs (solid gray bars) represents the normalized counts from the RNA-seq data and the right column of graphs (striped gray bars) represents the relative gene expression as determined by qPCR. Comp31514_c0_seq2, comp39985_c0_se4, and comp18014_c0_seq1 were annotated as the autophagy genes *PhATG6*, *PhATG8a,* and *PhATG8d*, respectively. Comp40361_c0_seq2 was annotated as Ein3-Binding F-box Protein 1 (*PhEBF1b*) and comp47181_c0_seq6 was annotated as Ethylene Insensitive 3-like Protein (*PhEIL1*).

To confirm the gene expression patterns identified by the RNA-seq data, the transcript levels of five genes were examined by quantitative PCR (Figure 7). Three of the genes (comp31514_c0_seq2, comp39985_c0_seq4, and comp18014_c0_seq1) were from Module III (grey60), which was characterized by higher expression at P18 compared to U18. Quantitative PCR analysis confirmed large differences in transcript abundance between P18 and U18, with much smaller changes between P12 and U12 and P24 and U24. Two additional genes that were identified as differentially regulated between pollinated and unpollinated libraries (P/U) by DESeq2 analysis (comp40361_c0_seq2 and comp47181_c0_seq6) also showed very similar patterns of transcript abundance as determined by RNA-seq and qPCR. All of the gene expression patterns were confirmed to be consistent with the RNA-seq data.

### Enriched GO terms suggest involvement of plant hormones within 12 hap

To identify the biological relevance of the pollination-associated gene changes, gene ontology (GO) was used to determine the biological processes, cellular components, and molecular functions of the differentially expressed genes [45] (Additional file 4). At 12 hap, 35 enriched GO terms were identified (FDR <0.05). Many of these terms involve plant hormones like abscisic acid (ABA), auxin, jasmonic acid (JA), and salicylic acid (SA). Of note are the response to auxin stimulus and response to 1-aminocyclopropane-1-carboxylic acid (ACC) GO terms. Both auxin and ACC are found in relatively high concentrations in pollen [42], and the corolla may be responding to hormonal signals that are transmitted through the gynoecium. At 18 hours after pollination, 154 enriched GO terms were identified including the ethylene signaling pathway. This coincided with the initiation of ethylene production from the corollas. Three of the molecular function GO terms involve autophagy. Autophagy is a catabolic process that involves transporting cellular components to the vacuole for further degradation and nutrient recycling [46]. No enriched terms were identified at 24 hap, but 368 enriched terms were identified when comparing pollinated to unpollinated (P/U) corollas at any time (12, 18, and 24 h). Enriched terms consisted of sucrose metabolic process, response to chitin, and response to wounding. The number of GO terms (557 in total of which 508 were unique) reflects the breadth of changes that occur between 12 and 24 hap in corolla tissue (Additional file 4).

### KEGG enrichment identifies pollination responsive pathways in the corolla

To identify the molecular pathways associated with pollination-induced corolla senescence, the significant DESeq2 and WGCNA genes were searched against *A. thaliana* proteins using BLASTx [47]. Top BLASTx hits were considered as the putative *A. thaliana* orthologs (Additional file 2). These hits were mapped to the Kyoto Encyclopedia of Genes and Genomes (KEGG) pathways. A total of 15 unique, enriched KEGGs were identified (Table 2 and Additional file 5). The KEGG pathways provided insight into potential biological pathways that function in the corollas of pollinated flowers. For example, eight of the KEGGs were involved in metabolism, including carbohydrate, energy, and lipid metabolism as well as the metabolism of terpenoids and polyketides. Other KEGGs were categorized under transport and catabolism, translation, signal transduction, and environmental adaptation.

**Table 2:** **KEGG enrichment hierarchy and mapping results**

**KEGG**	**Group** ^**b**^	**Mapped to KEGG**	**Total in KEGG**	**FDR** ^**a**^
**Metabolism**				
*Global and overview maps*				
Biosynthesis of secondary metabolites	18-P/U	123	551	3.12E-02
*Carbohydrate metabolism*				
Pentose and glucuronate interconversions	Module I	12	35	4.35E-04
Starch and sucrose metabolism	Module I	19	99	3.60E-03
Starch and sucrose metabolism	P/U	30	99	2.64E-02
*Energy metabolism*				
Carbon fixation in photosynthetic organisms	18-P/U	16	47	4.70E-02
*Lipid metabolism*				
Glycerolipid metabolism	18-P/U	15	39	3.12E-02
Alpha-Linolenic acid metabolism	18-P/U	9	20	4.70E-02
*Metabolism of terpenoids and polyketides*				
Limonene and pinene degradation	18-P/U	12	27	3.12E-02
*Biosynthesis of other secondary metabolites*				
Stilbenoid, diarylheptanoid and gingerol biosynthesis	18-P/U	12	30	4.50E-02
**Genetic Information Processing**				
*Translation*				
Ribosome	18-P/U	52	117	7.18E-10
Ribosome biosynthesis in eukaryotes	18-P/U	19	56	3.69E-02
**Environmental Information Processing**				
*Signal transduction*				
Plant hormone signal transduction	P/U	37	128	2.64E-02
**Cellular Processes**				
*Transport and catabolism*				
Endocytosis	Module III	15	70	3.20E-02
Peroxisome	Module III	13	55	3.20E-02
Peroxisome	18-P/U	18	55	4.70E-02
Regulation of autophagy	Module III	10	15	5.05E-06
Regulation of autophagy	18-P/U	9	15	1.42E-02
**Organismal Systems**				
*Environmental adaptation*				
Plant-pathogen interaction	Module I	20	92	5.46E-04
Plant-pathogen interaction	P/U	35	92	2.64E-02

^a^False discovery rate (*α* = 0.05).

^b^Modules were identified using WGCNA, P/U and 18-P/U were identified using DESeq2.

#### Four enriched KEGG pathways were identified in pollinated corollas

Four unique, enriched KEGG pathways were identified from the P/U genetic changes identified in DESeq2 and the WGCNA Module I (red). They included Plant-pathogen interactions, Starch and sucrose metabolism, Pentose and glucuronate interconversions, and Plant hormone signal transduction (Table 2). The genes within these KEGG pathways are associated with pollination and may contain key signaling components and molecular events that lead to flower senescence.

**Figure 8:**
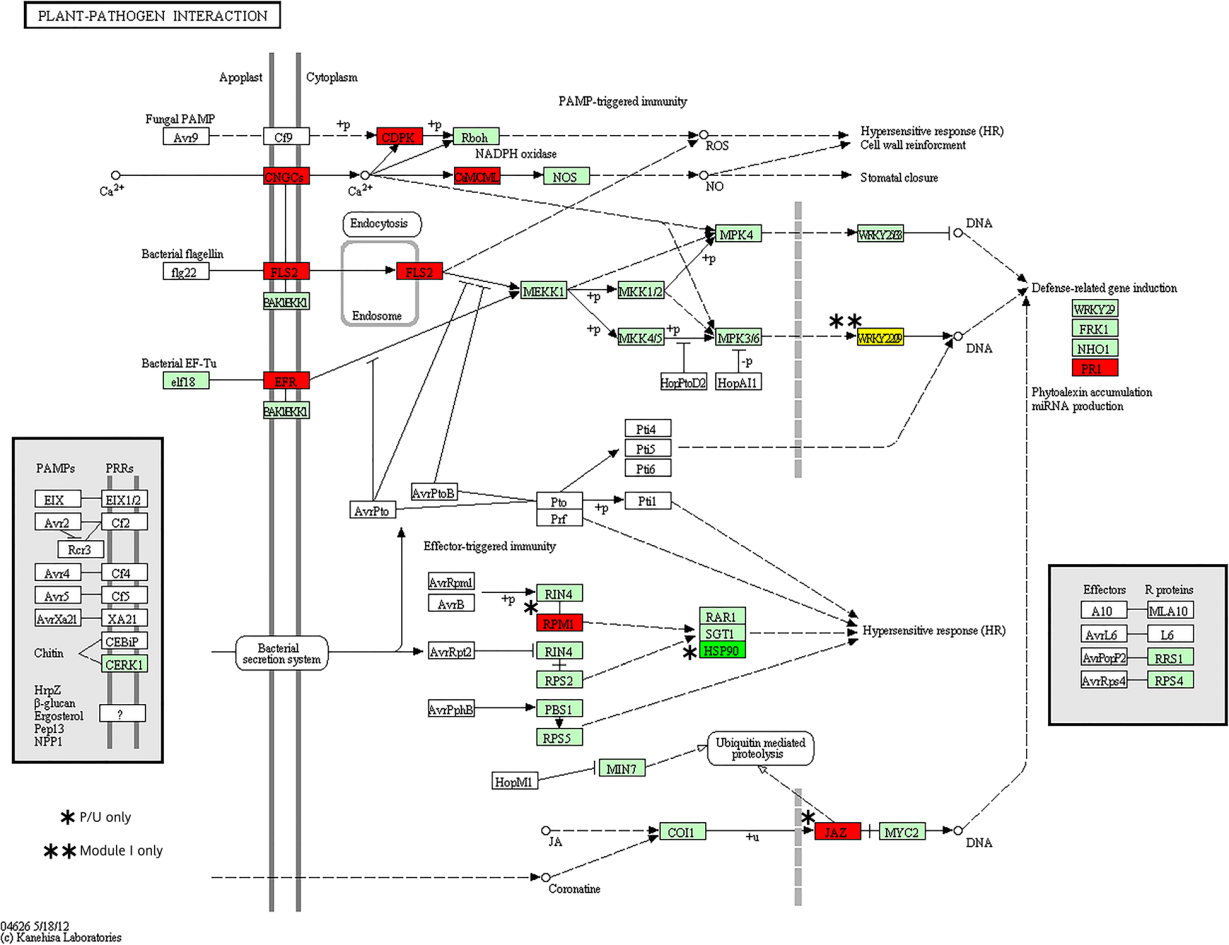
**Plant-pathogen interaction KEGG pathway.** TAIR codes from the top *A. thaliana* BLASTx hits of the differentially expressed P/U and Module I genes were mapped to the Plant-pathogen interaction KEGG pathway. Red boxes represent genes that were up-regulated in pollinated corollas, while dark green boxes represent down-regulated genes. Yellow boxes represent genes that were not found to be significantly differentially expressed but were grouped with Module I in WGCNA. Light green boxes represent *A. thaliana* genes that have been previously identified, while white boxes represent genes that belong to the KEGG pathway, but have no currently identified *A. thaliana* ortholog.

The Plant-pathogen interaction pathway was enriched in both P/U and in Module I. Genes encoding enzymes that are putatively involved in defense have been reported to be up-regulated during the senescence of many different flowers [16,23]. In our analysis, 35 P/U genes mapped to this pathway, and 20 genes mapped from Module I (Figure 8 and Additional file 5). The majority (80%) of these genes are predicted to interact with Ca^2+^ in this pathway, and some examples include calcium-dependent protein kinases, putative calcium binding proteins, and calmodulin-like proteins. While the role of calcium in the corollas of pollinated petunias has not been delineated, many studies have been performed to understand the role of Ca^2+^ signaling within the pollinated gynoecium. Changes in electrical potential have been observed [48], and calcium signaling is integral to pollen germination, pollen tube growth, and fertilization [49–51]. Our data suggest that Ca^2+^ signaling is continued from the style to the corolla and may be important for relaying pollination signals to the petals to initiate corolla senescence. A CBL-interacting kinase (CIPK) is up-regulated in an ethylene-dependent manner early in the senescence of carnation flowers [15]. CIPK regulates phosphorylation cascades that transmit Ca^2+^ signals, and it was hypothesized from these studies that calcium signaling was involved in carnation petal senescence.

Pollination and fungal infection share striking similarities in terms of biological responses, and both processes result in cell death [23,52]. X-ray microanalysis revealed that both pollen tubes and fungal hyphae penetration result in the accumulation of Ca^2+^ at the interaction sites [53]. Two well-known microbe-associated molecular pattern (MAMP) LRR receptor-like serine-threonine protein kinases, *flagellin insensitive 2* (*FLS2*) and *EF-Tu receptor* (*EFR*), were both up-regulated following pollination (Figure 8). Activation of these receptors results in changes in ion flux, reactive oxygen species formation, MAP kinase activation, and ethylene production [52]. It has been hypothesized that pathogen-related proteins are up-regulated during senescence to protect the senescing tissue from pathogenic attack [23], but petunia pollen tubes may contain an elicitor-like motif that activates FLS2 and EFR. Altering or eliminating these elicitors from pollen may prevent or delay pollination-induced senescence. Alternatively, increased expression of these genes may be a result of elevated ethylene levels. EIN3 and EIL have been shown to activate transcription of *FLS2* in *Arabidopsis* [54].

The Starch and sucrose metabolism pathway involves the catabolism of carbohydrates. The P/U list had 30 genes map to this KEGG pathway and Module I had 19 (Table 2 and Additional file 5). Many of these genes are involved in the conversion of UDP-D-galacturonate to D-galacturonate, which interacts with ascorbate metabolism. There are also many pectinesterase genes involved in the catabolism of pectin (Additional file 5). Soluble carbohydrates move from senescing to non-senescing flowers in gladiolus [55]. Sugars, particularly sucrose, increase in the phloem of *Ipomoea* and *Hemerocallis* (daylily) petals as the flowers open, mature, and senesce [56,57]. Labeling studies in carnations demonstrate that sucrose moves in the phloem from the petals to the gynoecium during senescence, and that this remobilization is accelerated by ethylene treatment [58]. The enrichment of this KEGG pathway suggests that a similar process involving the movement of carbohydrates to sinks, like the developing ovules, may also occur following pollination in petunia. Sucrose has profound effects on extending flower longevity, and has been implicated in the stability of EIN3 in *Arabidopsis* [59]. The application of sucrose to cut carnation flowers delays petal senescence and the up-regulation of genes involved in ethylene signaling [15]. The competition for carbohydrates also regulates the timing of senescence in ethylene-insensitive flowers like lilies (*Lilium*), where flower senescence is observed once the carbohydrate content of the tepals is reduced by ~50% [60].

The Pentose and glucuronate interconversions KEGG pathway was enriched in Module I, but not in the P/U gene list. A total of 12 mapped genes were found within Module I, nine of which involve pectin degradation (Table 2). This pathway contained five pectinesterase proteins, a polygalacturonase, and a UDP-glucose 6-dehydrogenase that overlap with the Starch and sucrose metabolism pathways. The other four Pentose and glucuronate interconversion-associated proteins are putative pectin lyase proteins. These enzymes are involved in cell wall loosening and have been shown to increase free Ca^2+^ levels as the calcium-cross-linked bridges are lysed (Additional file 5) [51,61,62]. Galactose loss is the main feature of cell wall changes during the senescence of petunia, *Sandersonia* and carnation flowers [63–66].

**Figure 9:**
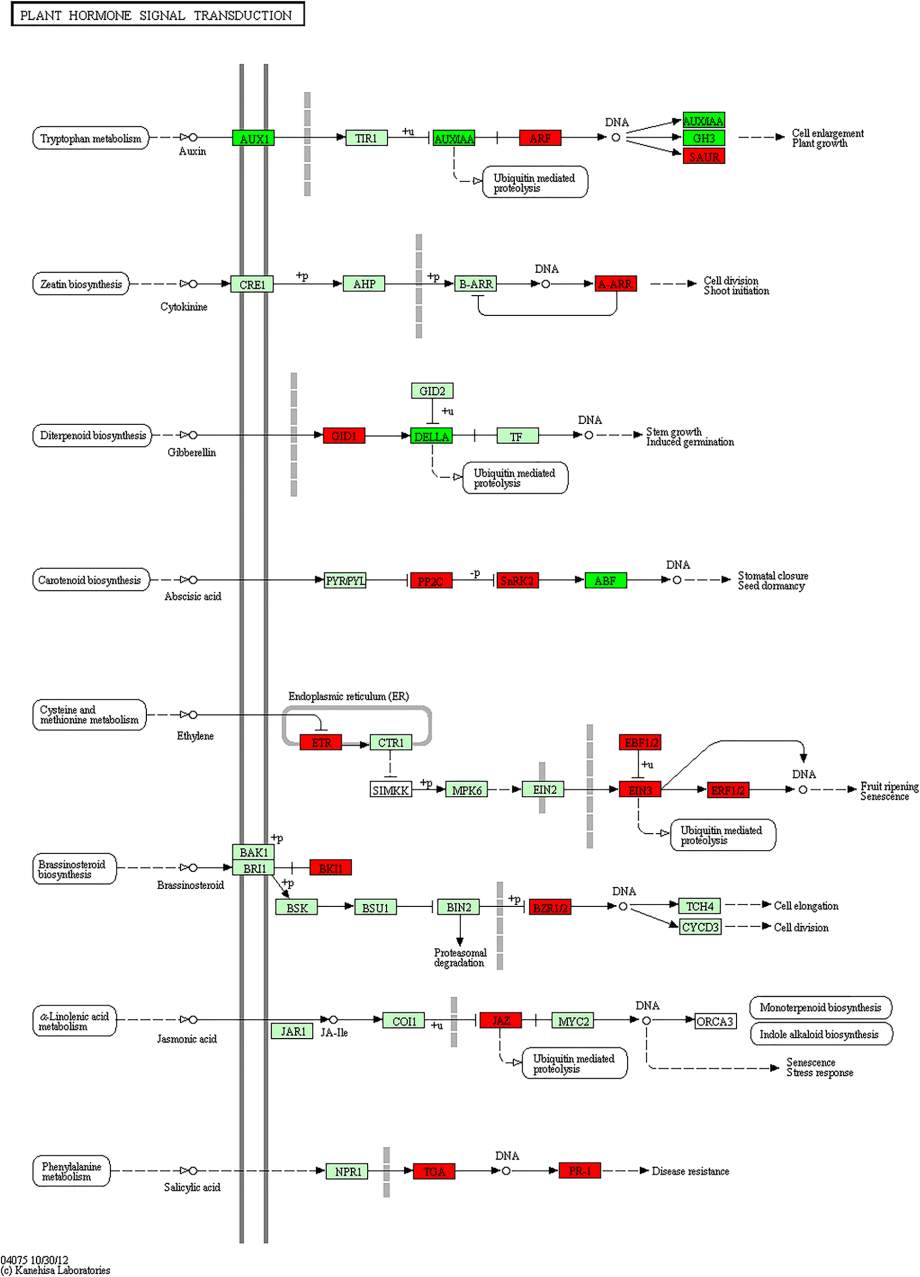
**Plant hormone signal transduction KEGG pathway.** TAIR codes from the top *A. thaliana* BLASTx hits of the differentially expressed P/U genes were mapped to the Plant hormone signal transduction KEGG pathway. Red boxes represent genes that were up-regulated in pollinated corollas, while dark green boxes represent down-regulated genes. Light green boxes represent *A. thaliana* genes that have been previously identified, while white boxes represent genes that belong to the KEGG pathway, but have no currently identified *A. thaliana* ortholog.

**Figure 10:**
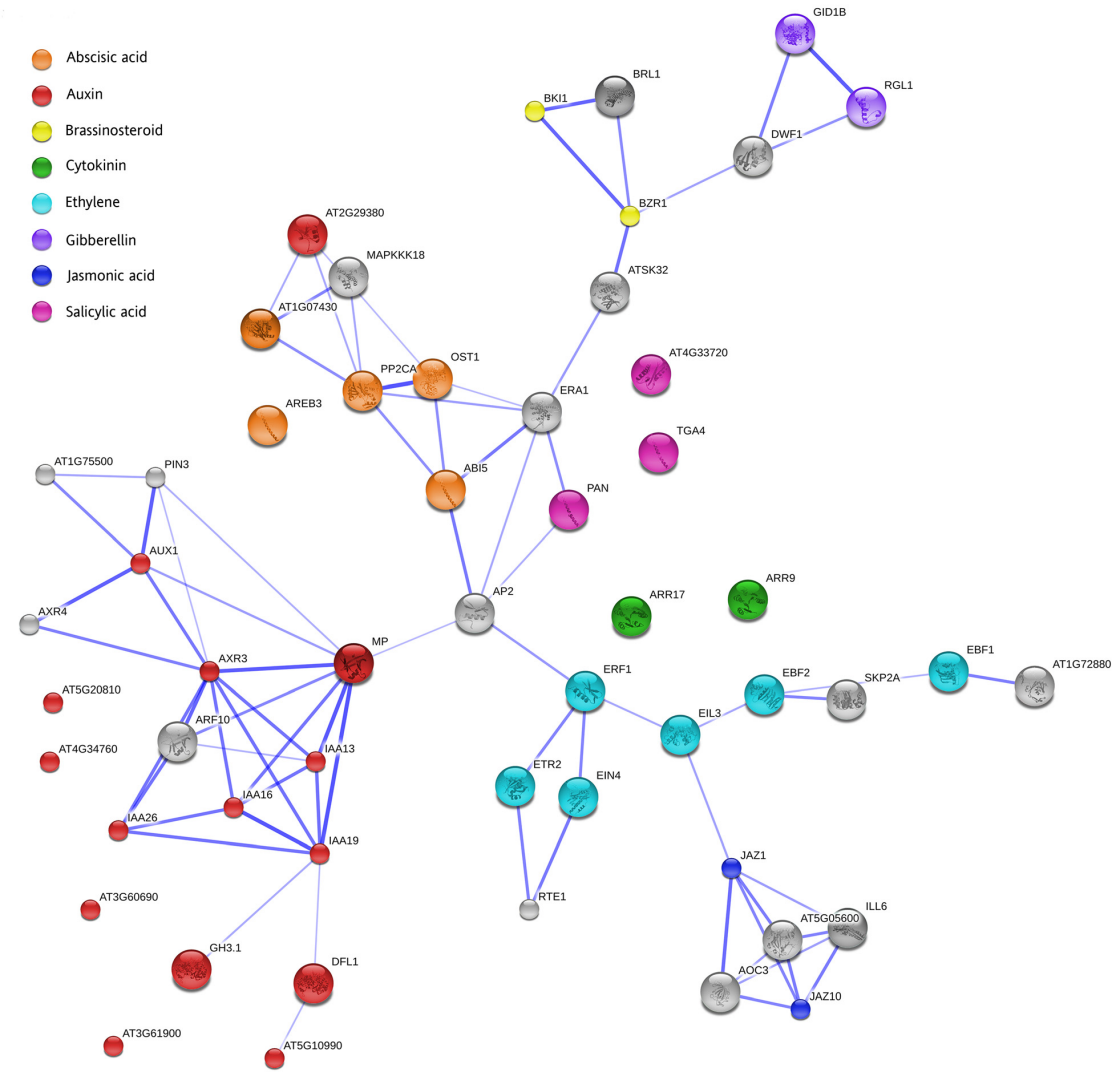
**STRING network of pollination-associated plant hormones genes.** Protein-protein interactions were graphed in STRING by inputting TAIR codes from the differentially expressed P/U genes that mapped to the Plant hormone signal transduction pathway and their nearest interacting partner (high confidence 0.700 threshold based on *A. thaliana* interactions). Colored bubbles correspond to the plant hormone and gray bubbles correspond to interacting partners that do not map to the Plant hormone signal transduction KEGG pathway. Edge thickness positively corresponds to the confidence of the interaction.

Plant hormones are an integral part of flower senescence [7], and the Plant hormone signal transduction KEGG pathway was enriched in the P/U gene list. Ethylene-sensitive crops, like petunia, produce ethylene after pollination [12], and the application of ethylene synthesis and perception inhibitors delays flower senescence when applied to the whole flower or to the pollinated gynoecium [43]. Transgenic petunias containing the mutant allele *etr1-1* [6] or a down-regulated *EIN2* [67] gene do not exhibit accelerated senescence after pollination, proving that ethylene is required for post-pollination signaling between the gynoecium and the corolla [4]. Nearly all of the 37 genes from the P/U gene list that mapped to this pathway were up-regulated, and they involved members of every major plant hormone pathway (Figure 9). Abscisic acid (ABA), ethylene, and jasmonic acid (JA) lead to genetic changes that promote senescence, while exogenous applications of cytokinin and gibberellin slow senescence [6,68–70]. The complex interplay of these plant hormones in petunia senescence is not fully understood, and protein-protein networks can provide preliminary information about potential targets for further analysis. We utilized STRING (string-db.org) to view the plant hormone protein interactions within a network using TAIR (The Arabidopsis Information Recourse) codes obtained via BLASTx restricted to *A. thaliana*. This network suggests that there are direct or indirect interactions between the plant hormones ethylene and JA as well as ABA and auxin (Figure 10). Of interest is the transcription factor APETALA 2 (AP2), which shares edges with ABA, auxin, ethylene, and salicylic acid (SA). This transcription factor belongs to the AP2/ERF family and is important for flower and seed development [71]. In *Brassica napus*, *BnAP2* RNAi lines have delayed flower abscission and senescence [72]. In tomato, *AP2* RNAi lines have altered levels of ethylene biosynthesis and differences in the timing of fruit ripening [73].

#### Eleven KEGGs are enriched at 18 hap

Large gene changes were observed specifically at 18 hap, and 11 enriched KEGG pathways were identified (see KEGGs designated with Module I and 18-P/U in Table 2). Most of the genes within the alpha-Linolenic acid metabolism, Endocytosis, Limonene and pinene degradation, Peroxisome, and Regulation of autophagy KEGG pathways were up-regulated following pollination, while the Carbon fixation in photosynthetic organisms, Ribosome, and Ribosome biosynthesis in eukaryotes KEGG pathways were down-regulated (Table 2, Additional file 5, and Additional file 6).

**Figure 11:**
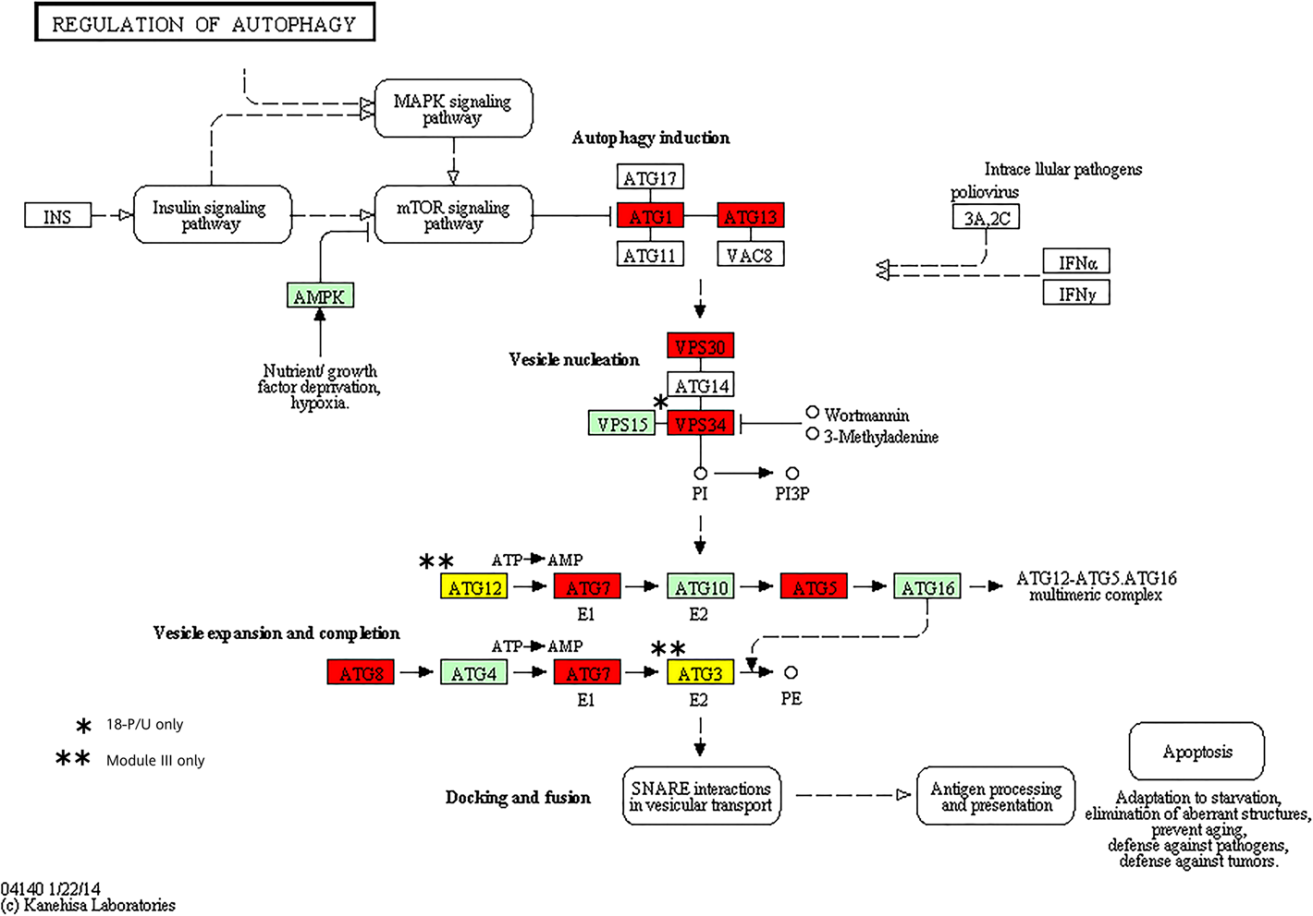
**Regulation of autophagy KEGG pathway.** TAIR codes from the top *A. thaliana* BLASTx hits of the differentially expressed 18-P/U and Module III genes were mapped to the Regulation of autophagy KEGG pathway. Red boxes represent genes that were up-regulated in pollinated corollas. Yellow boxes represent genes that were not significantly differentially expressed but were grouped with Module III. Light green boxes represent *A. thaliana* genes that have been previously identified, while white boxes represent genes that belong to the KEGG pathway, but have no currently identified *A. thaliana* ortholog.

The Regulation of autophagy was one of the most significantly (FDR = 5.05 × 10^−6^) enriched KEGG pathways, with 10 of 15 genes mapped. Autophagy is an intracellular degradation process where cytoplasmic constituents are transported to the vacuole for degradation so that nutrients can be remobilized [67,74]. Genes mapped throughout this pathway, including processes involving autophagy induction, vesicle nucleation, and vesicle expansion and completion (Figure 11). A previous high-resolution temporal profiling of Arabidopsis leaf senescence also identified 15 autophagy genes that were up-regulated during senescence [39]. Shibuya et al. [67] reported that the transcript abundance of *PhATG8* in petunia increases with ethylene exposure, and this coincides with increased nitrogen content within the ovary. The nitrogen content of ‘Mitchell Diploid’ petunia corollas decreases by greater than 60% during pollination-induced senescence [2]. Recently, pulse/chase experiments with ^15^ N have shown that nitrogen remobilization is reduced in *atg* mutants, and that this decreases biomass and yield [75,76]. Autophagy also clearly has a role in longevity, because *atg* mutants consistently display early leaf senescence. Evidence suggests that autophagy has both pro-survival and pro-death roles during plant development, but it is unclear how this dual function is regulated [46].

The petunia autophagy genes *APG5, APG7, APG8H, ATG1C, ATG13, ATG6, ATG8C*, and *ATG8F* are up-regulated at 18 h in corollas of pollinated flowers (Additional file 5). The 18-hour time point was collected after approximately 5 hours of darkness. This suggests that many pollination-induced autophagy genes may be regulated by darkness or may be functioning during the night. Rubisco degradation via autophagy occurs in early stages of dark-induced senescence [77]. During this process, Rubisco is remobilized to the vacuole, and a decrease in chlorophyll can be measured after just one day of darkness. However, in Arabidopsis *atg5* mutants, Rubisco is not remobilized in darkened leaves [74,77]. Changes in the expression of autophagy genes have also been reported during starch degradation in darkened Arabidopsis leaves [78]. Similarly, the remobilization of Rubisco from chloroplasts in the petunia corolla, which are primarily located in the tube, may be occurring via autophagy. Monodansylcadaverine (MDC) staining has been used to visualize the accumulation of acidic bodies during the pollination-induced senescence of petunia petals, but MDC staining is not specific to autophagosomes [67]. While all of these studies have provided compelling evidence for the involvement of autophagy in corolla senescence, additional morphological studies are needed to confirm the accumulation of autophagosomes in senescing petunia corollas [79].

### Five transcription factor families have more than 20 members in pollinated corollas

**Figure 12:**
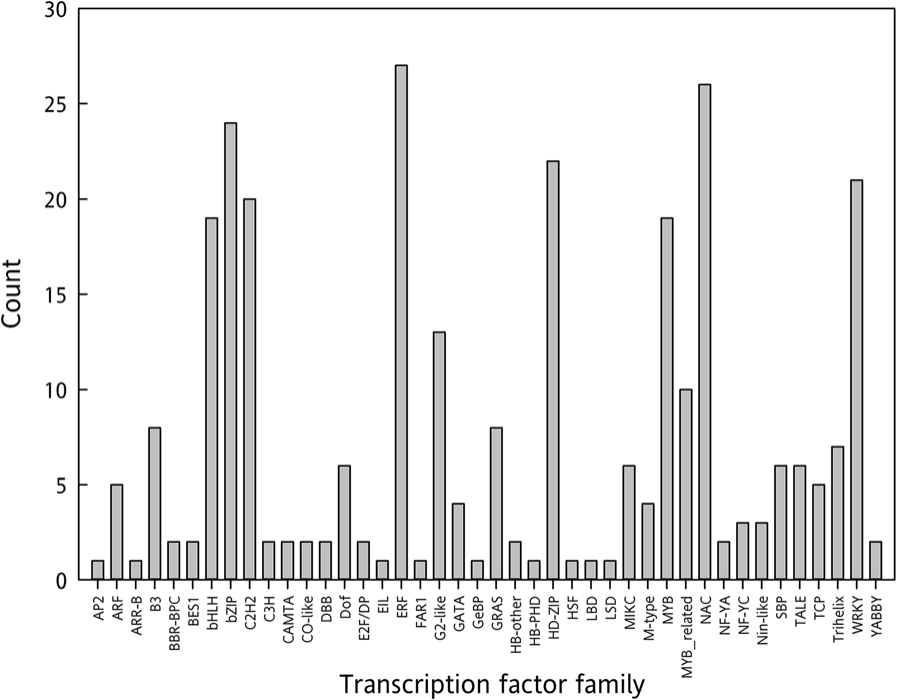
**Distribution of differentially expressed transcription factors.** Counts of transcription factors within the 4,746 differentially expressed genes were identified by searching the top *A. thaliana* BLASTx hits for TAIR codes within the *A. thaliana* transcription factor database (Plant TFDB v3.0).

Among the 4,746 differentially expressed genes we identified 301 putative transcription factors from 42 different families. More than 20 members from each of the following transcription factor families were identified: ERFs, NAC, bZIP, HD-Zip and WRKY (Figure 12). These transcription factor families have been implicated in senescence, abiotic stress responses, and plant hormone studies [80–84]. For example, in the bZIP transcription factor superfamily there is a subset of *Arabidopsis* transcription factors, termed S-type, that are master transcriptional and translational regulators of enzymes involved in amino acid catabolism under sucrose starvation and senescence-induced nutrient translocation [85,86]. In soybean (*Glycine max*) leaves, *GmbZIP53A* is up-regulated during sucrose starvation and may indirectly promote autophagy under those conditions [87]. As stated previously, sucrose has been shown to extend flower longevity and prevent ethylene production. In carnation, sucrose down regulates a key ethylene transcription factor *DcEIL3*, which is necessary for the initiation of the ethylene response [15]. Transcriptome changes in corollas from transgenic petunias with an inducible *etr1-1* were identified using microarrays [17]. This *etr1-1* transgene allows for ethylene insensitivity to be controlled by applying dexamethasone (DEX). A comparison between *etr1-1-*induced corollas and non-induced corollas revealed that B-box zinc finger, bHLH DNA-binding, homeodomain-like (HD), MADS-box, MYB domain, and NAC domain proteins are down regulated in ethylene insensitive corollas. These findings reflect our results, in that many of these proteins were found to be transcriptionally up-regulated after pollination (Figure 12). Altering the expression of transcription factors can have profound effects on flower longevity. For example, the down regulation of *PhHD-Zip* delays corolla senescence in both pollinated and unpollinated petunia flowers [83]. More research is needed to identify the functional role and interactions of these transcription factors for further manipulation of flower longevity.

### Uncharacterized genes may play an integral role in pollination and future research

The KEGG enrichment analysis provided a wealth of biological relevance through the identification of 15 uniquely enriched pathways. This provided a meaningful framework for the specific biological activities that are involved in pollination-induced corolla senescence in petunias. These enriched KEGG pathways still only represent a minority of the differentially expressed genes. The remaining differentially expressed genes likely also hold important biological relevance, particularly for those genes and pathways that might be unique to petunia. This analysis demonstrates the power of next generation sequencing to capture a global overview of thousands of gene expression changes in a single experiment. Understanding the relevance of these genes is currently the rate-limiting step. This technology provides fundamental data upon which more hypothesis-driven experiments can be organized and conducted.

## Conclusions

Pollination induces many hormonal, physiological, and molecular changes in petunia corollas that lead to senescence. Gene expression in the corollas was already altered by 12 hap, and 618 differentially expressed genes were identified. These changes occurred well before fertilization, ethylene biosynthesis from the corolla, and petal wilting. At 18 hap, large changes in gene expression were measured and an additional 2,137 genes were identified as being differentially expressed. The enriched GO term analysis suggested that at 12 hap, the corollas were responding to auxin and ACC, which are found in high abundance in pollen. KEGG enrichment identified 15 pathways, 11 of which were involved in metabolic pathways and autophagy regulation. The sequence data from this experiment will make a valuable contribution to the genomic resources available in petunia and will enable researchers to identify the genes involved in regulating flower senescence. While senescence studies have demonstrated that the initiation and timely progression of senescence requires transcription, senescence is also controlled post-transcriptionally. Previous studies in petunia have shown that genes expression patterns do not always correlate with protein expression [26]. Combined genomic, proteomic, and metabolomic approaches will be required to gain a comprehensive understanding of petal senescence.

## Methods

### Plant material

The plants used in this study were *Petunia × hybrida* (Hook.) Vilm. ‘Mitchell Diploid’ , a doubled haploid derived from a *P. axillaris*/*P.hybrida* ‘Rose of Heaven’ hybrid [88]. Seeds were originally obtained from Dr. David Clark (University of Florida). Petunia seeds were sown in plug trays on soil-less media (Pro-mix BX, Premier Horticulture, Quebec, Canada) and grown under fluorescent, full-spectrum lights. After four weeks, plants were transplanted into 16-cm pots and moved to a greenhouse with temperatures set at 24/16°C (day/night) and a 13 h photoperiod. Supplemental lighting was supplied by high pressure sodium and metal halide lights (GLX/GLS e-systems GROW lights, PARSource, Petaluma, CA, USA) to maintain light levels above 300 μmol m^−2^ s^−1^.

### Pollen tube growth measurements

To prevent self-pollination, anthers were removed 1 d before flower opening. On the following day, emasculated flowers were pollinated between 8:00 and 8:30 AM. Four styles were collected at 0, 12, 18, 24, and 36 hours after pollination (hap) and submerged in a 3:1 ratio of ethanol and acetic acid to fix the tissue overnight. They were then rinsed with 1 M potassium phosphate buffer (pH 7.0) followed by submersion in 1 N sodium hydroxide for 24 h. Finally, the styles were triple rinsed in sterile dH_2_O and stained with 0.1% aniline blue overnight. Styles were fixed on glass slides and visualized under an inverted epifluorescence Leica DM IRB microscope (Wetzlar, Germany) equipped with a Q Imaging Retiga 2000 cooled digital camera (Burnaby, BC, Canada). The lengths of the pollen tubes were measured using ImageJ [89].

### Ethylene measurements

Three biological replicates of two pollinated and unpollinated flowers were collected and photographed at 0, 12, 18, 24, 36 and 48 h. The corollas and styles from those flowers were collected and sealed in 22 mL and 7 mL glass vials, respectively. After a 30 minute incubation period, 1 mL of the headspace was withdrawn from each vial through a rubber septum in the lid. The samples were injected into a gas chromatograph equipped with a flame ionization detector and separated on a stainless steel column packed with HayeSep R (Varian 3800, Agilent, Santa Clara, CA, USA). The flow rate of the carrier gas (He) was 30 mL min^−1^.

### RNA extraction and library preparation

Flowers were emasculated as described previously. Four unpollinated and four pollinated flowers were harvested at 12, 18, and 24 h after flower opening. Three biological replicates were collected for each treatment-time combination. Corollas were detached from the receptacle (which removed the ovary and style), filaments were removed, and corollas were rinsed with sterile dH_2_O to remove any pollen contamination. Total RNA was extracted from the corollas using Trizol reagent (Invitrogen, Carlsbad, CA, USA) followed by an additional purification step using mini spin columns (Qiagen, Valencia, CA, USA). The quality of the RNA was determined using an Agilent 2100 Bioanalyzer RNA 6000 Nano kit (Agilent, Santa Clara, CA, USA) and it was quantified using a Qubit 2.0 fluorometer RNA Assay Kit (Invitrogen Inc. Carlsbad, CA, USA). A total of 5 μg of RNA was used to create each strand-specific RNA-seq library. Eighteen libraries (3 time points × 2 treatments × 3 biological replications) with six unique barcodes were prepared following the protocol of Zhong et al. [90], including the modification using the universal adaptor system. The libraries were sequenced at the Genomics Resources Core Facility at Weill Cornell Medical College (New York, NY, USA). Paired-end sequences (101 bp) were generated using three lanes of an Illumina HiSeq2000 flow cell (Ilumina Inc. San Diego, CA, USA). Individual biological replicates for each library were run in separate lanes on the flow cell.

### Sequence quality assessment and de novo assembly

Sequence qualities were assessed before and after trimming using FastQC version 0.10.1 (http://www.bioinformatics.bbsrc.ac.uk/projects/fastqc). Reads with a *Phred* quality score less than 20, and sequences shorter than 40 bp, were removed using trim-fastq.pl version 1.2.2 [91]. This resulted in two files that contained proper paired-end sequences and one file that contained sequences that lost the mate due to the preprocessing. Adaptors, barcodes, polyA, and polyT ends were trimmed using cutadapt version 1.2.1 [92]. After trimming, paired-end sequences were normalized to a maximum depth of 1,500 and assembled using Trinity r2012-10-05 [31]. To create an EST database for further analysis, the resulting contigs were screened for putative open reading frames (ORF) using the TransDecoder utility from Trinity. Additionally, contigs that had both an ortholog hit ratio [32] of more than 80% to the *Solanum lycopersicum* ITAG2.3 protein database (using BLASTx) and a unique component (comp#) and subcomponent (c#) were added to the EST library. Finally, CD-HIT-EST [93] was used to remove contigs that had 90% or greater sequence identity to each other.

### EST library annotation

The EST library was annotated using Blast2GO version 2.7.0 [94]. The translated Basic Local Alignment Search Tool (BLASTx) [47] was used to obtain top hits from the SwissProt database [95] for each contig using a minimum E-value threshold of 1.0 × 10^−3^. The remaining contigs with no BLASTx hits were aligned against the non-redundant (NR) database from National Center for Biotechnology Information (NCBI). Following the BLASTx step, finalized annotations for each gene were filtered with an E-value of 1.0 × 10^−6^, gene ontology (GO) terms were added, and conserved domains were identified using the InterPro Scan tool [96] for each contig. To obtain *A. thaliana*-specific annotations, the Arabidopsis proteome database was downloaded from UniProt and BLASTx was performed locally.

### Expression analysis from read mapping

Burrows-Wheeler Aligner (BWA) version 0.7.5a-r405 [97] was used to align the unprocessed Illumina reads to the EST library using the default alignment stringency. Paired-end and single reads that resulted from the pre-processing step as mentioned above were used to calculate the expression profile of each contig within a library. Sam2counts.py (https://github.com/vsbuffalo/sam2counts/blob/master/sam2counts.py) generated count tables of the reads that aligned to the EST library. Only uniquely-mapped reads were used for differential gene expression analysis. The R package DESeq2 version 1.4.1 [33] was used to determine the significant differentially expressed genes. In this package, principle component analysis (PCA) was used to screen for outliers among the libraries. A base mean threshold of ten was set to eliminate contigs with few counts, since contigs with very low reads typically have inaccurate expression patterns due to sampling error. Comparisons of all pollinated and unpollinated corollas (P/U), 12-h pollinated with 12-h unpollinated (12-P/U), 18-h pollinated and unpollinated (18-P/U), and 24-h pollinated and unpollinated (24-P/U) were made. An adjusted p-value (using the Benjamini & Hockberg adjustment) of 0.05 was used as the statistical cutoff for differentially expressed genes. A Venn diagram was used to visualize overlapping genes between comparisons [98].

### Quantitative PCR validation of gene expression patterns

The expression patterns of five genes (comp31514_c0_seq2, comp39985_c0_seq4, comp18014_c0_seq1, comp40361_c0_seq2, and comp47181_c0_seq6) were analyzed using quantitative real-time PCR (qPCR). RNA from four biological replicates of each treatment and time point were included in the qPCR analysis. cDNA was synthesized from 2 μg total RNA using Omniscript RT Kit (Qiagen, Valencia, CA). Primers were designed to amplify the specific transcripts using IDT Primer Quest (Additional file 7). Quantitative PCR was performed in a 20 μl reaction volume using iQ SYBR Green Supermix (Bio-Rad, Hercules, CA) with 1 μl cDNA as template as described previously [99]. Each reaction was performed in triplicate. Amplification specificity was determined by melt curve analysis. Amplification efficiencies of the target genes and reference genes were similar. Fold change for each target gene, normalized to *PhACTIN,* was calculated relative to expression in the U12 sample using the 2^-ΔΔ Cq^ method.

### Weighted gene correlation network analysis (WGCNA)

The R package WGCNA version 1.36 [34,35] was used to identify modules within the data set and to create dendrograms and heatmaps. A soft threshold value, power of 16, was used to transform the adjacency matrix to meet the scale-free topology criteria for optimal clustering. The libraries were clustered to identify outlier libraries using an average linkage hierarchical cluster tree based on Euclidean distance. Modules were grouped using a stringency threshold of 0.75. The code for the WGCNA analysis is available at the GetHub repository (https://github.com/wijerasa/WGCNA_Analysis). The pollination-specific modules were identified as Module I (red), Module II (cyan), and Module III (grey60) (Figure 5 and Additional file 3).

### GO and KEGG enrichment

An enriched GO term analysis was conducted using a Fisher’s Exact Test on the differentially expressed genes in Blast2GO. This test includes a correction for multiple testing [100] to reduce the false discovery rate (FDR). GO terms with a Term Filter Value of above 0.05 were excluded. TAIR codes for the EST library were obtained from a BLASTx that was restricted to *A. thaliana*. DESeq2 and WGCNA module contigs were mapped directly in KEGG mapper (http://www.genome.jp/kegg/mapper.html). The hypergeometric function in R was used to test for enriched pathways, and *P*-values were adjusted using an FDR correction.

### Protein-protein interactions visualized in STRING

To visualize the plant hormone protein interactions, TAIR codes for the P/U genes were uploaded into STRING to identify the genes belonging to the Plant hormone signal transduction KEGG pathway and their nearest interacting partner (stringency of interactions set at high confidence level of 0.70) [101]. The genes and their interacting partners were then uploaded into STRING and a network image was generated. The confidence view, which displays edges as blue lines, was selected and the image was exported. Colors of the circles were altered in Photoshop CS6 (Adobe, San Jose, CA, USA).

### Transcription factor analysis

Transcription factors within the 4,746 unique DESeq2 differentially expressed genes were identified by matching TAIR codes to the *A. thaliana* transcription factor database (Plant TFDB v3.0 [102]).

## Availability of supporting data

RNA-seq data were deposited with the Sequence Read Archive (SRA) database at NCBI (BioProject ID: PRJNA259884). Code for the WGCNA analysis can be accessed at the GitHub repository (https://github.com/wijerasa/WGCNA_Analysis).

## Additional files

Additional file 1:
**Biological replicate correlation scatterplots.** Pearson correlation coefficients (*R*) were calculated between the normalized count data from each (A) pollinated and (B) unpollinated biological replicate and graphed. The blue line represents the slope of the Pearson correlation. Additional file 2:
**Differential gene expression results and Module I and III annotations.** Differentially expressed genes were identified using pairwise comparisons (12-P/U, 18-P/U, 24-P/U and P/U) in DESeq2. Using BLASTx, gene annotation was obtained from the UniProt *A. thaliana* proteome. Each spreadsheet, which can be accessed through the tabs, contains the DESeq2 expression results for a single comparison, and two additional tabs contain the annotation results used for the KEGG enrichment of Modules I and III. Additional file 3:
**WGCNA cluster dendrogram.** Dendrogram of modules based on biweight midcorrelation calculations from variance stabilized count data generated by DESeq2. The colors of the dynamic tree cut correspond to the modules assigned for each gene. The merged dynamic colors display the assigned changes when the stringency threshold of 0.75 was used. Module I (red), Module II (cyan) and Module III (grey60) were identified as pollination-associated modules. Additional file 4:
**Gene ontology enrichment results of differentially expressed genes.** Gene ontology enrichment was performed on each list of differentially expressed genes from the DESeq2 pairwise comparison analyses (12-P/U, 18-P/U, 24-P/U and P/U). The enriched GO terms are organized by Biological Process, Cellular Component, and Molecular Function. Each spreadsheet, which can be accessed through the tabs, contains the GO term enrichment results for a single comparison. Additional file 5:
**Pathway mapping results for the genes belonging to the enriched pollination-associated KEGGs.** TAIR codes were obtained from the top *A. thaliana* BLASTx results for each DESeq2 differentially expressed contig and WGCNA pollination-associated module contig. Through hypergeometric testing, 13 enriched KEGG pathways were identified in the differentially expressed 18-P/U and P/U gene lists, and six enriched KEGG pathways were identified from the WGCNA pollination-associated modules. Each gene name corresponds with the adjacent *A. thaliana* TAIR code. The KEGG pathway codes and names have boldface type. Each spreadsheet, which can be accessed through the tabs, contains the enriched KEGG pathways belonging to the DESeq2 pairwise results (i.e. 18-PU and PU) or to the designated WGCNA module (i.e. Module I and Module III). Additional file 6:
**18-hour down-regulated KEGG pathways.** TAIR codes from the top *A. thaliana* BLASTx hits of the differentially expressed 18-P/U genes were mapped to the KEGG database and the (A) Ribosome KEGG pathway, (B) Carbon fixation in photosynthetic organisms KEGG pathway, and the (C) Ribosome biogenesis in eukaryotes KEGG pathway were found to be enriched. Red boxes represent genes that were up-regulated 18 hours after pollination in petunia corollas, while dark green boxes represent down-regulated genes. Light green boxes represent *A. thaliana* genes that have been previously identified, while white boxes represent genes that belong to the KEGG pathway, but have no currently identified *A. thaliana* ortholog. Additional file 7:
**Primers used for quantitative PCR.** Primers used to confirm expression patterns of select sequences from the RNA-seq analysis by quantitative PCR.
