# Supplementary figures and images for: RNA-sequencing reveals early, dynamic transcriptome changes in the corollas of pollinated petunias
Source: BMC Plant Biol. 2014 Nov 18;14:307. doi: 10.1186/s12870-014-0307-2 (PMC4245787; doi:10.1186/s12870-014-0307-2)

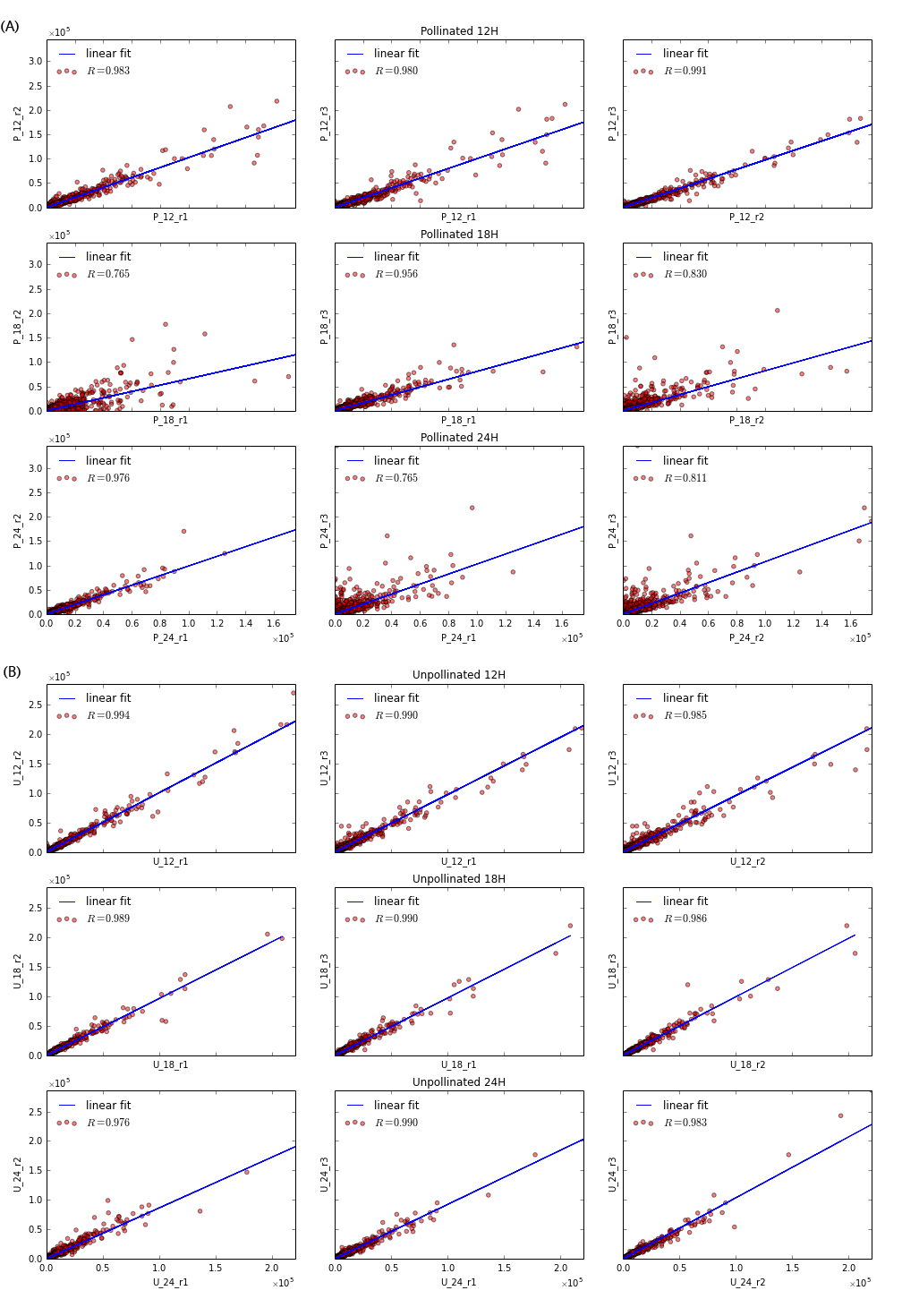

Supplement: Additional file 1: — Biological replicate correlation scatterplots. Pearson correlation coefficients (R) were calculated between the normalized count data from each (A) pollinated and (B) unpollinated biological replicate and graphed. The blue line represents the slope of the Pearson correlation. [file 12870_2014_307_MOESM1_ESM.tiff]

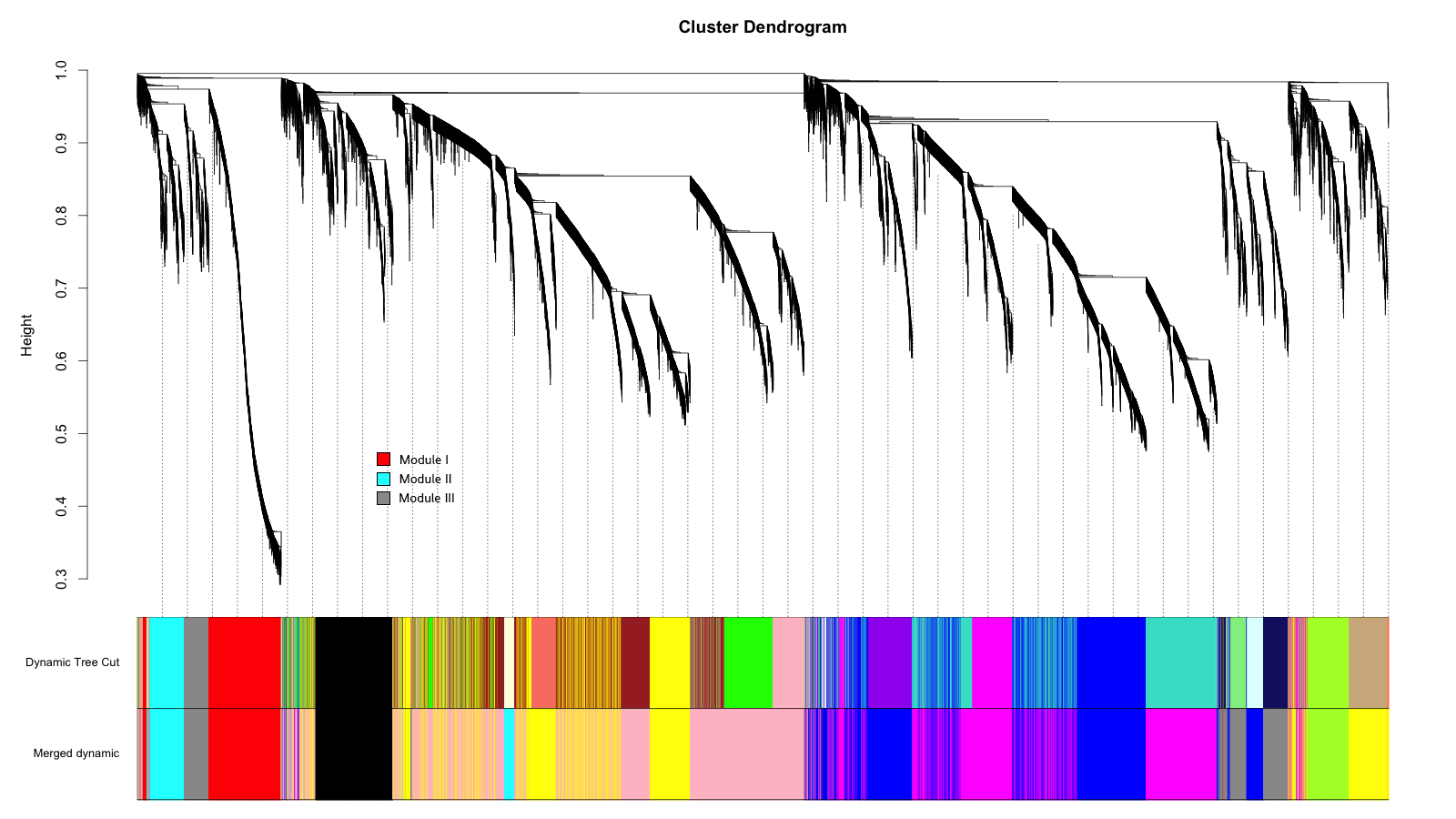

Supplement: Additional file 3: — WGCNA cluster dendrogram. Dendrogram of modules based on biweight midcorrelation calculations from variance stabilized count data generated by DESeq2. The colors of the dynamic tree cut correspond to the modules assigned for each gene. The merged dynamic colors display the assigned changes when the stringency threshold of 0.75 was used. Module I (red), Module II (cyan) and Module III (grey60) were identified as pollination-associated modules. [file 12870_2014_307_MOESM3_ESM.tiff]

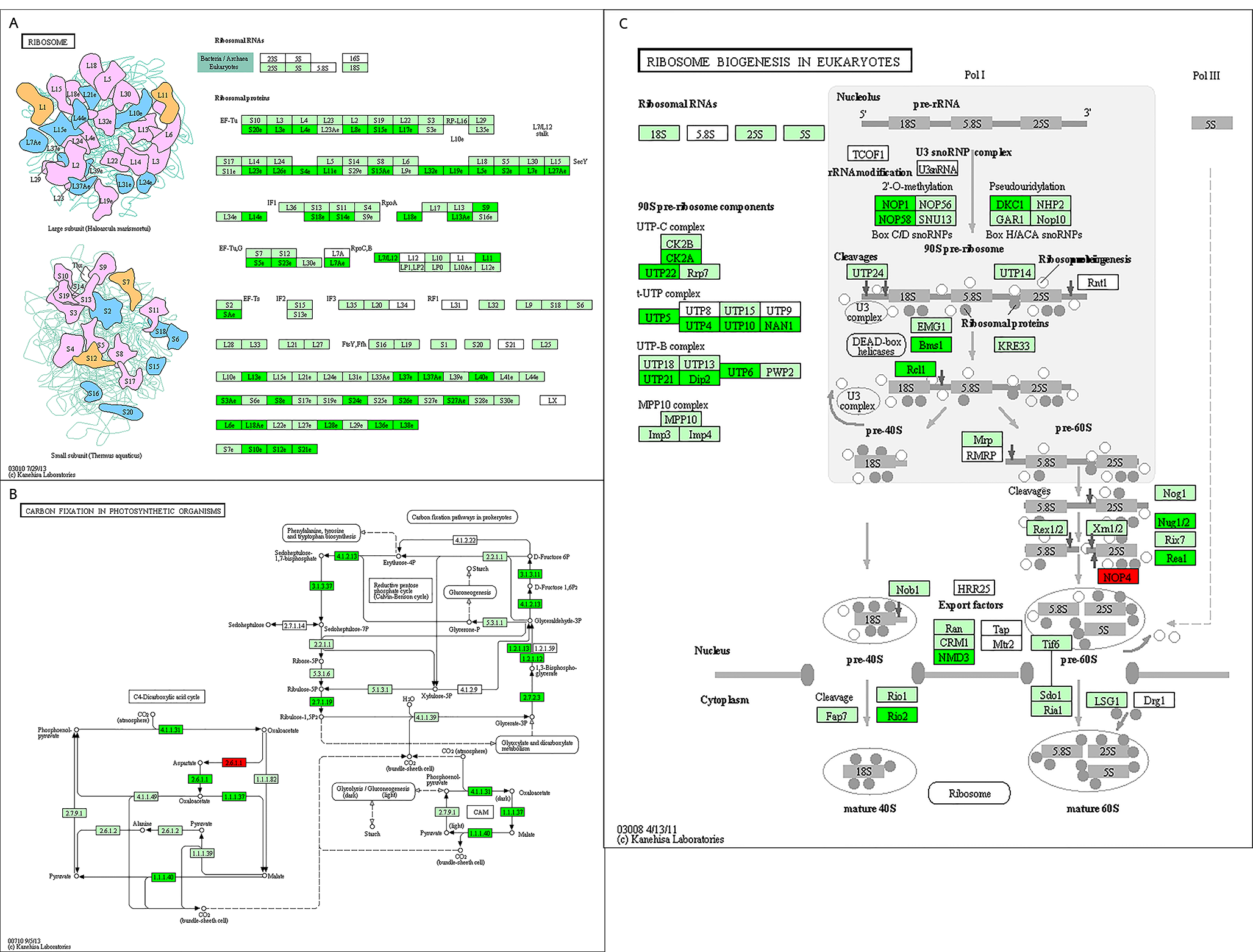

Supplement: Additional file 6: — 18-hour down-regulated KEGG pathways. TAIR codes from the top A. thaliana BLASTx hits of the differentially expressed 18-P/U genes were mapped to the KEGG database and the (A) Ribosome KEGG pathway, (B) Carbon fixation in photosynthetic organisms KEGG pathway, and the (C) Ribosome biogenesis in eukaryotes KEGG pathway were found to be enriched. Red boxes represent genes that were up-regulated 18 hours after pollination in petunia corollas, while dark green boxes represent down-regulated genes. Light green boxes represent A. thaliana genes that have been previously identified, while white boxes represent genes that belong to the KEGG pathway, but have no currently identified A. thaliana ortholog. [file 12870_2014_307_MOESM6_ESM.tiff]
