# Supplementary material for: RNA-sequencing reveals early, dynamic transcriptome changes in the corollas of pollinated petunias
Source: BMC Plant Biol. 2014 Nov 18;14:307. doi: 10.1186/s12870-014-0307-2 (PMC4245787; doi:10.1186/s12870-014-0307-2)
Supplement: Additional file 7: — Primers used for quantitative PCR. Primers used to confirm expression patterns of select sequences from the RNA-seq analysis by quantitative PCR. [file 12870_2014_307_MOESM7_ESM.pdf]

Additional file 7 Primers used for quantitative PCR. Primers used to confirm expression patterns of select sequences from the RNA-seq analysis by quantitative PCR.

**Primer Name**	**Primer Sequence (5’ – 3’)**	**Corresponding Sequence ID**
F *PhACTIN* RT	AGCCAACAGAGAGAAGATGACCCA	CV299322
R *PhACTIN* RT	ACACCATCACCAGAGTCCAACACA	
F *PhATG6* RT	GAGCGAGCAGCAATTTTAGC	comp31514_c0_seq2
R *PhATG6* RT	CACTCGACCGGAATCTTAGG	
F *PhATG8a* RT	TGGCAGACAGCTGACTTTTC	comp39985_c0_seq4
R *PhATG8a* RT	TTTTAAACAACCATTTGAAGAGA	
F *PhATG8d* RT	TTGCATGCATCCTCACTCTT	comp18014_c0_seq1
R *PhATG8d* RT	TCAGTTAAAGGGTATAGACAAATCCA	
F *PhEBF1b* RT	CTCCGAAATTTGCAGGAGAGA	comp40361_c0_seq2
R *PhEBF1b* RT	AGCAGCAAGTCTAACATCAGTAG	
F *PhEIL1* RT	GCAAGCTGCTGAGAAGCAGAACAA	comp47181_c0_seq6
R *PhEIL1* RT	TCCGATGCACCACTCACAGGTTTA	
